# Indifferent minds, broken system: a critical examination of mental health care provision for Spain’s incarcerated population with serious mental illnesses

**DOI:** 10.3389/fpsyt.2024.1340155

**Published:** 2024-08-21

**Authors:** Alfredo Calcedo-Barba, Joaquín Antón Basanta, Silvia Paz Ruiz, Alvaro Muro Alvarez, Edorta Elizagárate Zabala, Verónica Estévez Closas, Angeles López López, Luis Fernando Barrios Flores

**Affiliations:** ^1^ Institute of Psychiatry and Mental Health, Gregorio Marañón General University Hospital, Medical School, Complutense University of Madrid, Madrid, Spain; ^2^ Spanish Society of Legal Psychiatry, Madrid, Spain; ^3^ General Practice Penitentiary Health Care, Albolote Penitentiary Centre, Granada, Spain; ^4^ Spanish Society of Penitentiary Health, Barcelona, Spain; ^5^ SmartWorking4U, Valencia, Spain; ^6^ Penitentiary Psychiatry Services, Barcelona, Spain; ^7^ Psychiatry Service of the Mental Health Network of Araba, Mental Health Centre, Zaballa Penitentiary Center, Spanish Society of Clinical Psychiatry, Deusto University Medical School, Bilbao, Spain; ^8^ Retired, Alicante, Spain

**Keywords:** mental healthcare models, prisons, mental healthcare resources, serious mental illness, mental healthcare provision

## Abstract

**Background:**

Spain healthcare system is decentralized, with seventeen autonomous regions overseeing healthcare. However, penitentiary healthcare is managed nationally, except in Catalonia, the Basque Country, and Navarra. These variations impact mental health care provision for inmates with serious mental illness (SMI).

**Objective:**

To delineate differences between regions in terms of mental health care provision for individuals with SMI, available resources, and the perspectives of healthcare professionals operating in the Spanish prison environment.

**Methods:**

Employing an explanatory sequential mixed-method approach, the study conducted an extensive literature review, quantitative data collection through structured questionnaires, and qualitative data collection via focus groups and four in-depth interviews. Analysis involved calculating percentages and ratios for quantitative data and thematic analysis for qualitative data interpretation to comprehensively understand mental healthcare provision.

**Results:**

In December 2021, about 4% of inmates in Spain had SMI. There are three distinct models of mental healthcare within the Spanish prison system. The traditional penitentiary model, representing 83% of the incarcerated population, operates independently under the General Secretariat of Penitentiary Institutions at a national level. This model relies on an average of 5.2 penitentiary General Practitioners (pGP) per 1,000 inmates for psychiatric and general healthcare. External psychiatrists are engaged for part-time psychiatric assessment. Acute psychiatric hospitalization occurs in general nursing modules within penitentiary centers or in Restricted Access Units (RAUs) in reference hospitals. Two penitentiary psychiatric hospitals provide care to unimputable SMI inmates from all over Spain. Innovative penitentiary models, constituting 17% of the prison population, integrate penitentiary healthcare within regional public health systems. The Basque Country features a Mental Health Unit with full-time care teams within the penitentiary center. Catalonia emphasizes community care, providing full-time dedicated psychiatric services within and outside prisons, ensuring continued care in the community. Both models prioritize personnel with specialized mental health training and compensation akin to non-prison healthcare settings.

**Conclusions:**

Regional disparities in penitentiary mental healthcare models in Spain result in resource inequalities, impacting specialized care for inmates with SMI and opportunities for healthcare professionals. The models in the Basque Country and Catalonia offer valuable experiences for penitentiary healthcare.

## 1 Introduction

The occurrence of mental illnesses among incarcerated individuals ranges from as low as 2% to as high as 48% in various studies (1). In most cases, this rate surpasses the estimated prevalence of these disorders in the general community. In the challenging prison environment, individuals grappling with serious mental illness (SMI) confront heightened vulnerability (1). This vulnerability stems from the debilitating psychiatric symptoms they often endure, compromised overall health, and significant limitations in their social functioning, necessitating support for basic daily tasks. SMI is a practical classification based on clinical diagnosis, lasting over two years, and the presence of disability, whether functional or intellectual (2, 3) This classification empowers healthcare professionals to identify individuals most in need and tailor mental health interventions accordingly (4).

Spain comprises 17 autonomous regions, each with the authority to oversee its healthcare system and provide healthcare services to residents under the public National Health System. However, penitentiary healthcare has remained under the jurisdiction of the General Secretariat of Penitentiary Institutions at the national Ministry of Interior, a non-health care orientated institution, in most regions, except Catalonia, the Basque Country and Navarra. This raises concerns, particularly when one considers that prisons are not designed to function as clinical treatment facilities and lack the adequate expertise, infrastructure and funding to provide the comprehensive care necessary for individuals with SMI (1, 5).

Spanish overarching legal framework guarantees inmates the same health rights and healthcare services, including pharmaceutical and non-pharmaceutical benefits, as the general population outside prison walls. It stresses the importance of healthcare objectives, the proportionality of security measures, and the desirability of delivering care and treatment as close to the inmate’s home community as possible (6–9).

In 2003, the Cohesion and Quality of the National Health System Law mandated the crucial transfer of penitentiary healthcare responsibilities from the Ministry of the Interior’s General Secretariat of Penitentiary Institutions to regional governments (10). This transfer aimed to integrate these healthcare services into regional healthcare systems, covering the healthcare needs of all residents in the regions under the public National Healthcare System. Regions were provided with the option to decide when to assume these responsibilities within an 18-month period (10). As of 2023, only Catalonia, the Basque Country, and Navarra have embraced these roles. The other 14 regions are not yet in compliance with the law and the General Secretariat of Penitentiary Institutions at the national level remains responsible for the psychiatric and overall healthcare of inmates.

Given this uneven landscape, regional differences determine the disparate organization, structure, and management of healthcare services within prisons, impacting the most vulnerable groups, including inmates with SMI. This research seeks to answer the question: How is healthcare provision for incarcerated individuals with SMI managed in Spanish penitentiary facilities? It aims to describe regional differences in healthcare provision for individuals with SMI, available resources, and the perspectives of healthcare professionals operating in the prison environment. Additionally, it seeks to showcase the achievements attainable by integrating penitentiary healthcare provision into regional healthcare systems, highlighting two innovative models in the Basque Country and Catalonia that may serve as references for future endeavors.

## 2 Materials and methods

An explanatory sequential mixed-method approach was employed. Mixed-methods research is an investigative technique in which a researcher or a team combines elements of both qualitative and quantitative research methods, including qualitative and quantitative perspectives, data collection, analysis, and inference techniques (11).

The research process involved three main steps: a literature review was conducted to establish the current knowledge on the topic; structured questionnaires were used to collect quantitative data from the General Secretariat of Penitentiary Institutions regarding healthcare resources; and qualitative data was gathered through focus groups with healthcare professionals involved in SMI care within penitentiary settings, followed by in-depth interviews with experts from regions that had transitioned penitentiary healthcare responsibilities from national to regional governments. It is important to note that Navarra is included in the group of regions that have not yet assumed responsibility for their penitentiary healthcare. As of the time of reporting these research findings, the traditional model of penitentiary healthcare provision still prevails, as Navarra only acquired these responsibilities in 2021.

**Supplementary Figure 1** illustrates the research methodology used.

### 2.1 Literature review

An extensive and organized narrative review of the literature was conducted to comprehensively depict the current state of healthcare for individuals with SMI in Spanish penitentiary facilities (12). This review encompassed both indexed literature and gray literature, serving as the foundation for the research thesis and helping to identify gaps in existing knowledge.

For indexed literature, searches were conducted on databases like PubMed, Scopus, Google Scholar, MEDES, and Dialnet, targeting relevant publications in both Spanish and English (**Supplementary Table 1** and **Supplementary Figure 2** provide search details). In the gray literature, data was collected from official Spanish and international websites, with a particular emphasis on European sources offering content in Spanish or English. Additionally, non-indexed specialized electronic journals were consulted, conference publications were reviewed, and both indexed and gray literature were manually searched to supplement the insights gained from focus groups and expert interviews.

The extensive and organized narrative review of the literature conducted helped to identify and summarize diverse sources, which may not have been captured in a systematic review. In these cases, a comprehensive narrative review allows for the flexibility to include diverse sources, explore the complexity of the topic, and achieve a more comprehensive understanding (13). Due to the diverse nature of the publications, the comprehensiveness of the review, and the fact that literature was also sought to contrast and compare findings in the research, it is not possible to state the final number of publications retrieved and reviewed. This approach aligns with the recommendations for comprehensive literature searches, which emphasize the importance of thorough and systematic searches to minimize bias and ensure comprehensive coverage of the research topic (14).

The review uncovered inconclusive evidence in several areas, including the availability of healthcare resources, the profile and prevalence of individuals with SMI in penitentiary centers, the presence of specialized care resources, drugs consumptions, and the care trajectory for inmates requiring mental health services both within and outside prison. While pockets of knowledge existed regarding best practices and effective care models, notably the Catalan penitentiary mental health care model and the developing penitentiary mental healthcare unit in the Basque Country, their visibility within the scientific literature remained limited. To address these knowledge gaps, focus groups were conducted, expert interviews were held, and additional data was obtained from the Spanish General Secretariat of Penitentiary Institutions through a structured questionnaire (see **Supplementary Figure 1**).

### 2.2 Quantitative data collection

The quantitative data presented in this research was obtained from various sources. This included referring to the annual reports published by the General Secretariat of Penitentiary Institutions, which contained data up to 2020 and had been updated in 2021. Additionally, other reports from the same institution were consulted as they were published in response to specific inquiries from the Spanish Parliament and were made available in the Official Gazette of the *Cortes Generales*.

Furthermore, a structured questionnaire was employed to request supplementary data from the General Secretariat of Penitentiary Institutions. This questionnaire focused on human and healthcare resources, as well as drug usage in penitentiary centers in regions where penitentiary healthcare management had not been transferred from the national to the regional authorities. The data request was for the year 2020 and was submitted electronically through the transparency portal of the General State Administration.

### 2.3 Qualitative data collection

#### 2.3.1 Focus groups

Five focus groups were conducted between May 31 and July 19, 2022. Each group consisted of 2 to 6 health professionals, totaling 11 primary care physicians and psychiatrists, 4 nurses, and 3 pharmacists who provide daily healthcare in Spanish prisons, and care for inmates with serious mental health problems. Focus groups allowed to gain insight into participants’ perspectives, attitudes, beliefs, and opinions on mental healthcare provision for seriously ill inmates (15, 16).

Multiple focus groups were conducted to ensure information saturation, a point at which recurring data no longer adds interpretive value or when emerging theories adequately explain collected data (17). Three to six different focus groups, or the same meeting several times, are sufficient to achieve information saturation in research contexts like this (18).

A limited number of participants were chosen for each focus group (intentional sampling), with selection criteria based on their specialized knowledge and professional experience within the specific domain of penitentiary healthcare (17). Invitations were extended to the medical director of all 71 Spanish penitentiary centers, and those who responded affirmatively (n=23) were invited to contribute to this study.

Invitations were sent via email, and all participants who accepted (n=18) took part in focus groups lasting between 100 and 153 minutes. Two participants were located in the Basque Country, while the remaining focus group interviewees worked in regions that did not have penitentiary healthcare responsibilities at the time of the study. Participants were categorized based on two criteria: either they belonged to a large (> 1000), medium (450-1000), or small (< 450) penitentiary center according to the number of detainees in their respective centers, or they were associated with the penitentiary psychiatric hospitals. Participants from Catalonia preferred to provide written information and declined to participate in the focus groups.

To guide these focus groups, a set of open-ended questions was used to address various topics, including the specific care needs of inmates with SMI, human and structural care support resources available for penitentiary and community penitentiary healthcare, everyday mental healthcare provision.

#### 2.3.2 In-depth interviews

The primary objective of the in-depth interviews was to gain a comprehensive understanding of good practices and innovative models for the provision of mental healthcare identified in the literature review and focus groups. They had been implemented in specific penitentiary settings in concrete regions (19–21). Key experts responsible for these initiatives were selected and invited to participate in two-hour interviews, which were audio-recorded and transcribed.

To guide these interviews, the researcher used a set of open-ended questions that focused on various aspects of the practice or model core characteristics. These questions covered topics such as the model’s inception, the needs it addresses, the care it provides, the political and financial context in which it operates, its structure, and organization, the profile of beneficiaries, factors contributing to its success, areas for improvement, and prospects.

### 2.4 Data analysis

#### 2.4.1 Quantitative data

Due to limited and sometimes inconsistent data, percentages were used to describe aspects of prison populations, and no additional scales or statistical tests were applied to summarize the data. Data on the total prison population by region and penitentiary center was primarily derived from 2020 figures with updates through 2021 (see **Supplementary Table 2** for source details). Information about inmates with high mental healthcare needs was obtained from the PAIEM (*Programa de Atención Integral al Enfermo Mental*) program as of July 2019, the latest publicly available data (22). Calculations for assessing the relationship between individuals with SMI and the total prison population by region were based on 2019 data (see **Supplementary Table 3**).

Data on structural healthcare resources within penitentiary facilities, including the number of nursing module beds and the availability of Restricted Access Units (RAU) in referral hospitals, were extracted from the 2020 and 2021 General Reports of the General Secretariat of Penitentiary Institutions (23, 24). Ratios of these resources per 100 incarcerated individuals were calculated using 2020 data, as specified in **Supplementary Table 3**. The Reports of the General Secretariat of Penitentiary Institutions, issued annually, provide an overview of facilities, resources, and the health situation of the incarcerated population in Spain. However, the data is reported in aggregated form, limiting its interpretation and research usability. Additionally, the National Institute of Statistics (INE), which provides data on the total incarcerated population size grouped by regions, gender, type of offense, and sentence, was consulted in this study to contrast findings (25). The Report of the General Secretariat of Penitentiary Institutions was chosen for consistency.

Ratios assessing the availability of physicians working in prisons for 2020 were estimated using data obtained from the General Secretariat of Penitentiary Institutions in response to a public information request (see **Supplementary Table 2**) (26). Additionally, ratios of penitentiary General Practitioners (pGP) per 1000 inmates, categorized by region, were calculated for 2018, 2019, and 2021 following the methodology outlined in **Supplementary Table 3**, with data sources referenced in **Supplementary Table 2**.

The percentage of medical personnel who retired for various reasons, including voluntary retirement or age-related factors, relative to the total medical staff, was calculated based on annual reports from the General Secretariat of Penitentiary Institutions for 2018, 2019, 2020, and 2021 (23, 24, 27, 28).

The ratio of psychiatrists specializing in mental health per 1,000 inmates at each penitentiary center was based on 2019 data obtained from a parliamentary response (29). The percentage of full-time dedication among psychiatrists was estimated using input from prison healthcare professionals in focus groups. Estimates of psychiatric healthcare resource utilization in 2020 were derived from data provided by the General Secretariat of Penitentiary Institutions (26, 30, 31), covering specialized psychiatric consultations, admissions to nursing modules for psychiatric pathologies, and hospital discharges following admission to reference hospitals for mental disorders, relative to the total available healthcare resources.

#### 2.4.2 Qualitative data

The analysis of both the focus groups and the individual interviews involved a team of three researchers. Initially, two researchers independently analyzed the transcripts, and any discrepancies in their analyses were resolved by the third researcher. To facilitate this process, the researchers utilized the MAXQDA^®^ tool, which provided support for coding, analysis, and synthesis of the transcripts.

##### 2.4.2.1 Focus groups

The focus group sessions were recorded, transcribed, and analyzed using a deductive-inductive approach following the constant comparison thematic analysis technique (32). This technique allowed for assessing information saturation across multiple focus groups, revealing recurring themes with subtle variations based on the penitentiary center’s size to which participants were affiliated (17).

##### 2.4.2.2 In-depth interviews

Thematic analysis of the in-depth interview transcripts was conducted with a deductive approach (32). The findings from the in-depth interviews encompassed themes such as the Extended Bridge (*Puente Extendido*) program, deprescribing practices, rational medication usage, innovative and comprehensive mental healthcare provision models in the Basque Country and Catalonia, respectively. This paper focuses on mental healthcare provision models existing in the Spanish penitentiary setting, with subsequent publications addressing good practices like deprescribing and rational medication use in prisons.

### 2.5 Ethical aspects

The research protocol received approval from the Research Ethics Committee at Ramón y Cajal University Hospital on February 22, 2022 (MINUTES 428).

## 3 Results

### 3.1 The Spanish incarcerated population

**Figure 1:**
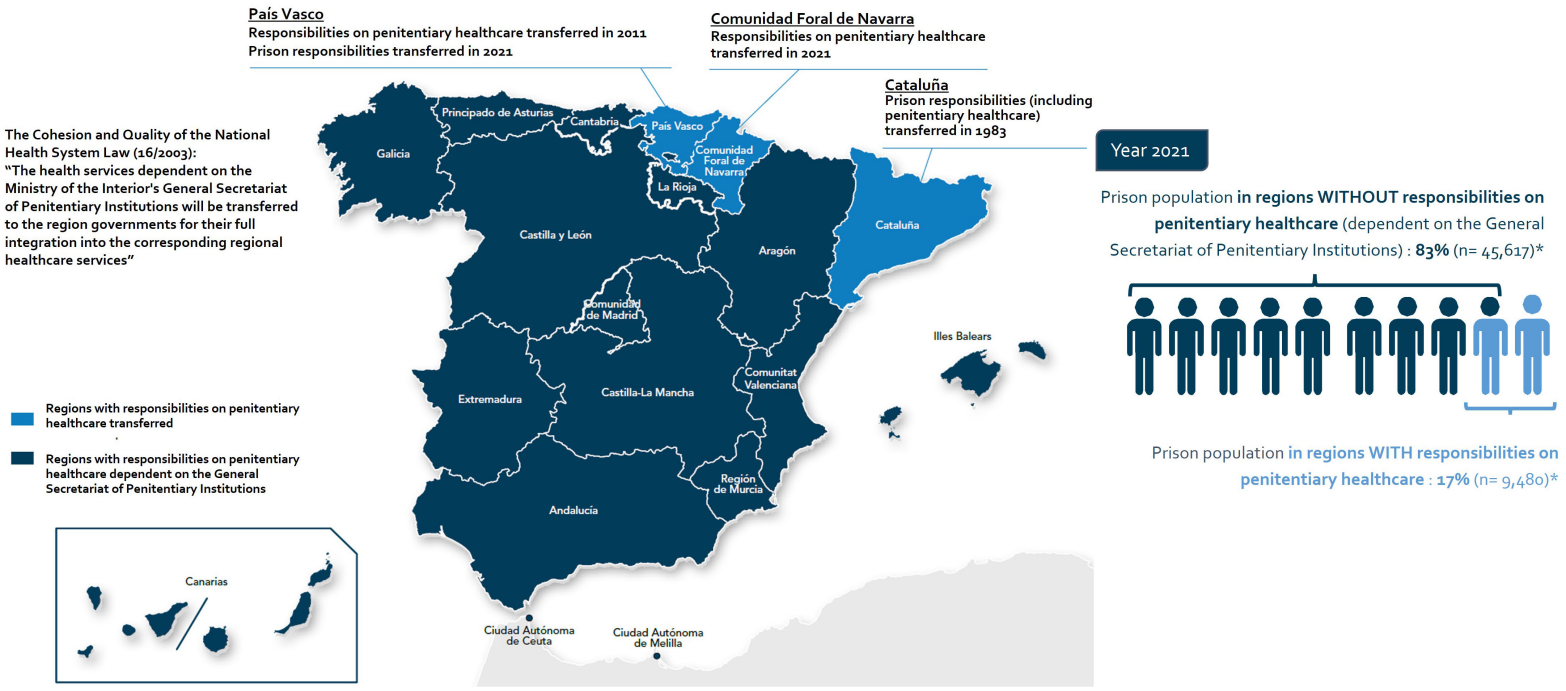
Distribution of the prison population in penitentiary centers in regions with and without responsibilities on penitentiary healthcare provision in Spain. * Estimate made based on the three regions with transferred prison healthcare responsibilities, using as prison population data those corresponding to the year 2021, extracted from the National Statistical Data Report on the prisoner population for December 2021 issued by the General Secretariat of Penitentiary Institutions (33). For the preparation of this map, the Spanish official names of the regions were used.

Official data from December 2021 revealed that Spain’s total prison population stood at 55,097 inmates, which included pre-trial detainees. Among them, 45,617 individuals (83.0%) were under the jurisdiction of regions still managed by the General Secretariat of Penitentiary Institutions (33) (**Figure 1**). Women comprised 7.1% of the overall inmate population in ordinary Spanish prisons (33). In Spain, the majority of women were incarcerated in mixed penitentiary centers (34, 35).

These numbers remain very similar in 2023 representing a prison population rate of 113 per 100,000 inhabitants based on an estimated national population of 48.06 million at beginning of January 2023 (36).

The distribution of the incarcerated population varied across regions, as did the available resources for their care. In 2020, the General Secretariat of Penitentiary Institutions oversaw a total of 66 ordinary penitentiary centers (excluding Catalonia and the Basque Country). The most populous regions, Andalusia, the Community of Madrid, and the Valencian Community, had the highest number of incarcerated individuals and the most prisons (26) (**Supplementary Table 4**).

### 3.2 The Spanish incarcerated population with SMI

Although several epidemiological studies have sought to assess the prevalence of individuals with SMI in Spanish prisons (**Supplementary Table 5**) determining its precise percentage poses a challenge due to data limitations (37).

Considering 2021 estimates from the General Secretariat of Penitentiary Institutions, around 4% of inmates in Spanish penitentiary centers had SMI. This estimate took into account the number of individuals participating in the PAIEM that registered 1,834 inmates during that year (**Supplementary Table 4**) (24). Based on PAIEM participation data, SMI was present in 90.2% of male inmates and 9.8% of female inmates (23, 24). Their distribution varied across Spanish regions, with Andalusia (24%) and the Valencian Community (20%) reporting the highest percentages (22).

The most common diagnoses were psychotic (32%), dual (a mental health disorder associated with a substance use disorder (SUD), 27%), and affective disorders (14%) (23). Personality disorders accounted for 21% of all diagnoses (24, 38). More than one third of those with SMI required a high level of assistance due to reduced autonomy (23, 24) (**Supplementary Table 6**).

### 3.3 The traditional mental healthcare model for the incarcerated population with SMI in regions without penitentiary healthcare responsibilities

In 14 out of 17 regions (all regions with the exception of the Basque Country, Catalonia and Navarra), healthcare provision in the penitentiary setting remains dependent on the General Secretariat of Penitentiary Institutions and operates as a parallel, separated system with limited integration into the National Healthcare System (39, 40) (**Figure 1**).

**Figure 2:**
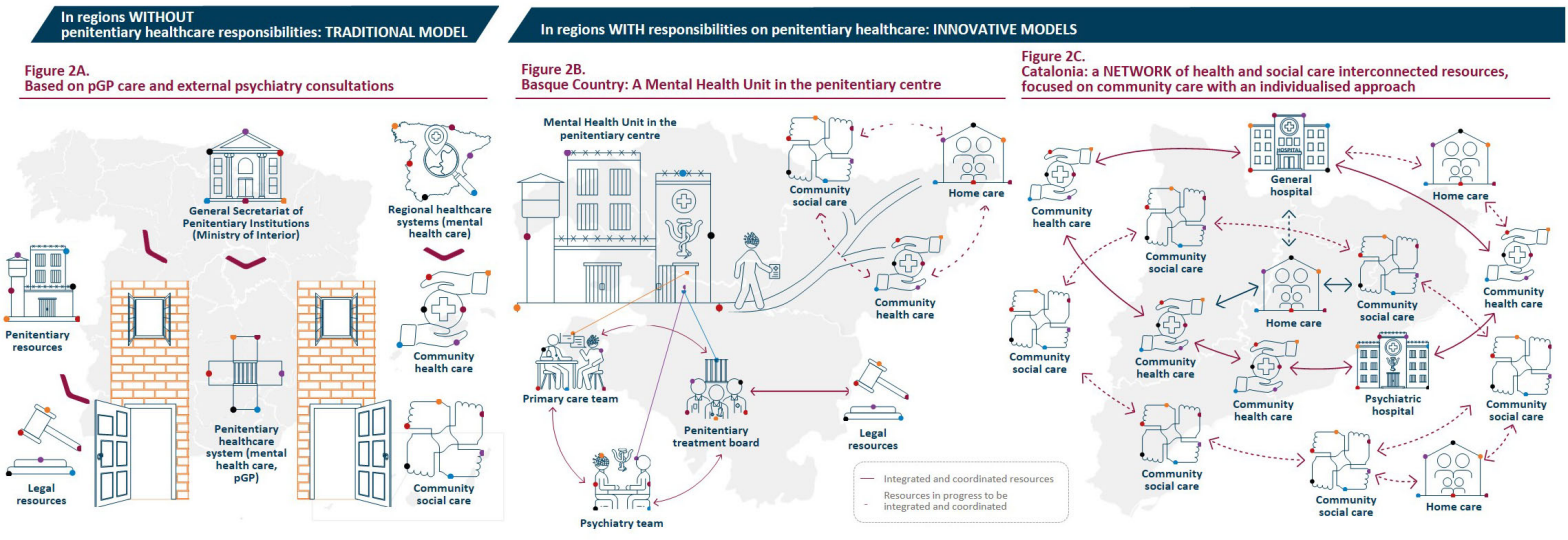
Models for mental healthcare provision in penitentiary centers in Spain. **(A)** in regions without penitentiary healthcare responsibilities, a traditional model prevails in which the penitentiary healthcare system operates independently and in parallel with the regional healthcare systems. Health care is mostly provided by pGP and access to specialized healthcare, including psychiatric care, is significantly compromised in this scenario. **(B, C)** in regions with penitentiary healthcare responsibilities, innovative models have emerged. The integration of the penitentiary healthcare system into the regional healthcare systems has improved the availability and access to healthcare resources, promoting coordinated services to ensure adequate psychiatric care. pGP, penitentiary general practitioner.

The traditional penitentiary healthcare model delivers primary care using resources within the penitentiary institutions. Specialized healthcare, on the other hand, draws upon resources from the National Healthcare System outside of prisons (39, 40) (**Figure 2A**). Psychiatric hospitalization takes place in two penitentiary psychiatric hospitals situated in Alicante and Seville, which serve the entire Spanish unimputable population with SMI outside Catalonia and the Basque Country. Due to limited capacity, individuals judicially involved and diagnosed with SMI requiring psychiatric care and treatment are admitted to ordinary penitentiary centers. Here, psychiatric care is provided by part-time, external consultants who visit inmates in prisons while acute hospitalizations mostly take place in general nursing modules within the penitentiary center or at general referral hospitals (41) (**Supplementary Table 4**).

#### 3.3.1 General practitioners in the traditional penitentiary healthcare model

In 2020, ordinary penitentiary centers had an average of 5.2 pGP per 1,000 inmates (23), lower than the World Health Organization (WHO) European region average of 8.0 (42). Although some regions exceeded the WHO average, most of them had ratios at or below 7 pGP per 1,000 inmates. The ratio was lower in larger penitentiary centers (2.0 - 6.3 pGP per 1,000) compared to the smaller ones (4.0 - 21.7 pGP per 1,000) (**Supplementary Table 7**). In over 40% of the ordinary penitentiary centers, medical staff primarily worked on-call, with no in-person duties unless deemed necessary (26).

Moreover, between 2018 and 2020, the ratio of primary care General Practitioners (pGPs) per 1,000 inmates declined from 6.1 to 5.2. This decrease primarily stemmed from limited recruitment of professionals, resulting in a more pronounced reduction in medical personnel compared to the growth of the prison population. This trend persisted into 2021 (23, 24, 27, 28, 43). Healthcare professionals who participated in the focus groups corroborated these findings, which are also documented in the literature (44, 45) (**Tables 1**, **2**). Challenging working conditions, lack of professional recognition and incentives, and uncertainty regarding career advancement prompt many to explore alternative career paths outside of correctional facilities. With a relatively small penitentiary healthcare workforce, replenishing it poses significant challenges, leading to fewer professionals bearing heavier workloads (44, 45).

**Table 1:** Challenges in managing SMI in central penitentiary administration’s ordinary prisons: insights from healthcare professionals in focus groups.

Key issue	Description	Illustrative Quotes
**Early detection of undiagnosed mental disorders**	High rate of undiagnosed conditions	*“More than 60% come to us undiagnosed, they have already been through prisons (…)”* [G1; 00:26:35.860].
**Psychiatry care in penitentiary ordinary centers**	Challenges in communication and availability	*“Many times, we go through our personal telephone numbers, to ask [the psychiatrist] questions or to talk about a patient that we want to refer to the hospital. (…) The problem is that (…)[the psychiatrist] is not present in the center for a long time”* [G2; 01:14:44.130].
**Management of exacerbations of psychiatric symptoms**	General practice training and handling emergencies	*“We [General Practice] physicians are normally well trained because we have been there for many years and we are trained to handle a psychiatric emergency typically”* [G3; 00:38:46.690].
	Frequent hospital transfers due to lack of on-site physicians	*“It’s a rare week that we don’t have to take someone to the hospital because there is no way to stabilize them. Often, we lack a doctor who can assess whether the patient needs to remain here, and in such cases, we prefer to transfer them out”* [G3; 00:51:21.260].
	Managing care on-site with available physicians:	*“It’s quite unusual for us to transfer someone out or send them to the acute unit. We typically manage their care ourselves”* [G3; 00:37:57.310].
**Management of mental disorders at referral general hospitals**	Hospital admission challenges	*“Initially, our admissions were not accepted at the hospital because it did not meet the requirements for accommodating both the police and inmates. As a result, they would return them almost the next day”* [G1; 01:04:06.510].
	Lack of custody facilities	*“We lack a custody unit and a waiting room in the hospital, so our inmates are treated like the general population. This situation poses significant challenges for both the police and the hospital”* [G2; 01:15:48.660].
**Pharmacological treatment for mental disorders**	Initial approach and patient engagement	*“We don’t start with depot formulations right away because patients usually resist it. We first attempt to engage in a dialogue and reach an agreement on the treatment plan. Typically, we start with oral formulations, and if necessary, we transition to depot formulations over time”* [G1; 01:23:23.240].
	Effectiveness of depot medications	*“We currently have many treatments that are, that are parenteral, that are depot, which are administered every month, and that stabilizes them quite a bit”* [G3; 00:10:24.260].
	Ensuring adherence	*“We often use depot medication, mainly to ensure adherence”* [G1; 01:22:09.150].
**Daily monitoring and inmates support**	Reliance on inmate cooperation	“*If the inmates themselves didn’t assist, it would be entirely impossible [to manage inmates with SMI], as there are insufficient resources to manage numerous tasks without their involvement”* [G3; 00:18:04.960].
**Continuity of care upon transfer or release**	Lack of access to medical records post-transfer and after release	*“After the patient is discharged from the center, even if they are transferred to another penitentiary facility, we no longer have access to their medical records”* [G2; 01:05:48.130].
	Challenges with therapeutic community referrals	*“Individuals referred to therapeutic communities often face expulsion due to rule violations, resulting in a potential return to prison due to new criminal activities”* [G1; 01:29:32.190].

SMI, Serious Mental Illness.

**Table 2:** Unmet needs in penitentiary psychiatric hospitals under the central penitentiary administration in Spain: insights from healthcare professionals in focus groups.

Unmet need	Overarching need	Illustrative quotes
**Organization**	Peer review by equally qualified professionals	“*Given what a psychiatrist decides, at a given moment, we can question many things, but I believe that it can only be questioned by someone of equal standing who possesses the same training, not by someone lower in professional expertise*” [01:08:14.].
**Staffing**	Unsustainable burden of sole responsibility	“*The question is, how long will that last? Because for almost three years, I had the disastrous experience of being the only psychiatrist in the center. And that is absolutely unbearable; I was accumulating days, or vacations, whatever was necessary*” [02:06:39.380].
**Workload**	Overwhelming lack of support	“*I was alone for seven months, (…) I was alone for the entire hospital. That includes seeing patients, making reports. I have made more than 100 reports, that is, having them accumulated because I couldn’t keep up*” [02:10:40].
**Conflicts of interest**	Challenges of patient honesty and dual roles	“*You see a patient that you can examine, then you treat him/her, and then every six months you inform the judge whether the patient leaves or not. Sometimes (the patient) has very little desire to tell you that he/she is unwell because he/she knows that after six months you are going to tell the judge if he/she can leave or not*” [02:15:15.040].
**Professional incentives**	Disparity in compensation and career progression	“*We don’t have a professional career; we earn less [than psychiatrists/physicians working in the community]. You really have to be very ‘vocational’ to be in the center*” [02:06:39.380].
**Retirement coverage**	Impending shortage of primary care physicians	“*As for Primary Care physicians, currently, we have three, but one is about to retire soon, and two more are on the verge of retirement. Next year, they will all retire, leaving none behind*” [02:13:52.390].
**Specialized training in Psychiatry**	Lack of specific training profiles	“*Training profiles are not requested for individuals working in the penitentiary psychiatric hospital*” [00:54:27.600].
	Distinct role of surveillance officers	“*The role of a surveillance officer in a prison psychiatric facility cannot be the same as that in a standard correctional center due to the unique characteristics and idiosyncrasies that define these distinct roles*” [01:14:54.990].
	Need for psychiatric expertise in leadership	“*A psychiatric hospital cannot be effectively managed by a medical director or deputy medical director who lacks a psychiatric background*” [00:58:51].
**Psychiatry training supervision**	Concerns about psychiatry resident supervision	“*In the teaching committees, our instructors who mentor our psychiatry residents are often doctors with a specialization in Family and Community Medicine. It appears ethically questionable to me that psychiatry in-training doctors would be supervised by a professional who lacks expertise in psychiatry and meets with them only three or four times a month, if at all*” [00:58:51].

#### 3.3.2 Psychiatrists in the traditional penitentiary healthcare model

**Figure 3:**
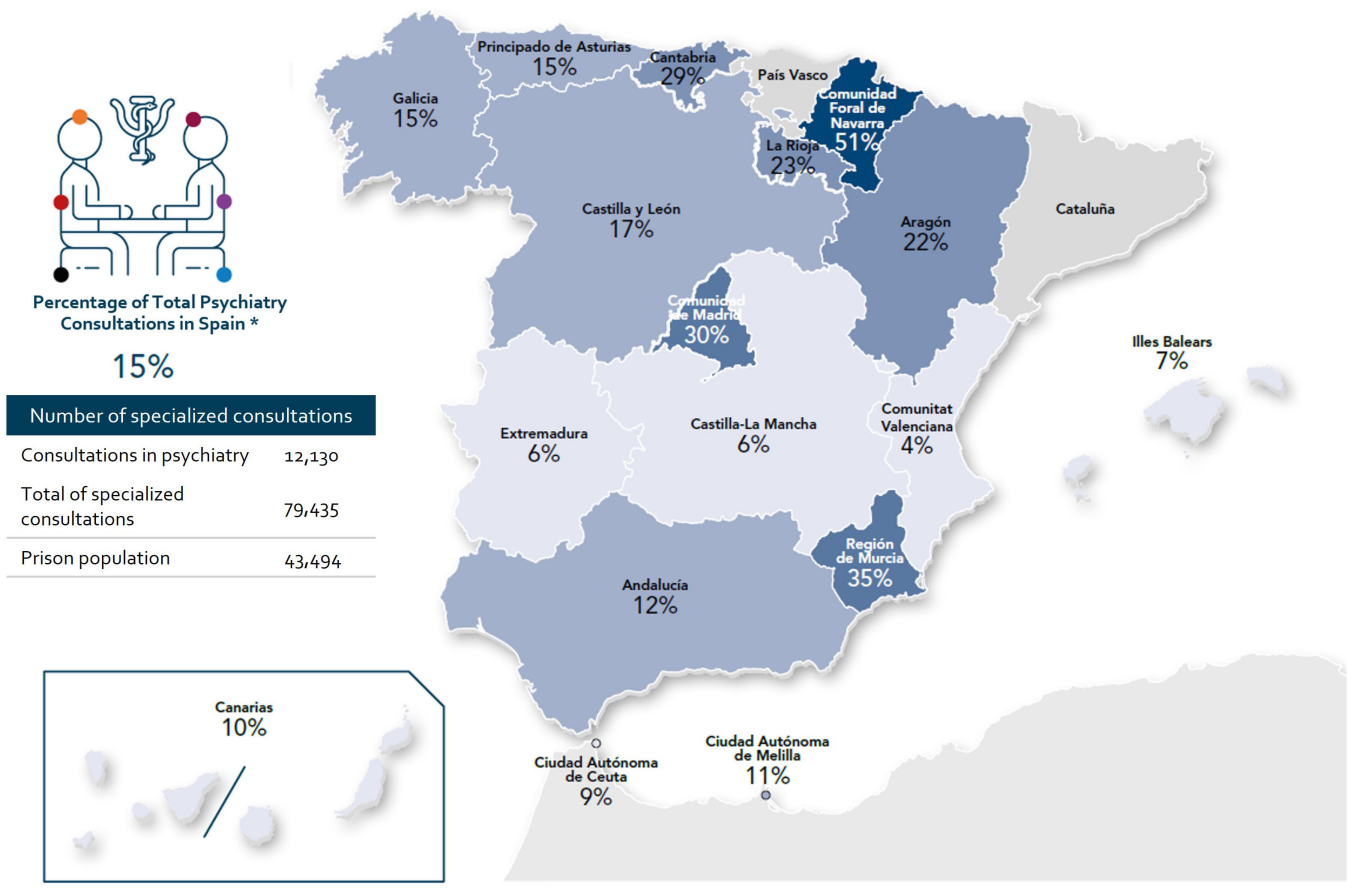
Percentage of psychiatry consultations compared to the total number of specialized consultations in regions without penitentiary healthcare provision responsibilities in 2020. *Percentages calculated from the data on the number psychiatry consultations by regions and total number of specialized consultations by regions*100, extracted from the report generated by the Secretariat General of Penitentiary Institutions (Ministry of the Interior) in response to the request for transparency made by Dr. A. Calcedo, on September 5, 2022 (Annex I Specialized care: global and centers2020 (30). These data do not include the population of the Penitentiary Psychiatric Hospitals of Alicante and Seville. Important note: All presented data are estimates of a specific moment that pretend to reflect the status of the situation and trends at the time of data collection. Data should be interpreted with caution, and the status should be verified at each point in time. For the preparation of this map, the Spanish official names of the regions were used.

Ambulatory specialized psychiatric care within ordinary penitentiary centers ruled by the General Secretariat of Penitentiary Institutions relies on psychiatrists working part-time as referral consultants under various contractual arrangements (**Supplementary Table 4**). Psychiatric care was available in 95% of ordinary penitentiary centers, and out of these, 61% (38 out of 62) were provided by psychiatrists affiliated with public healthcare services (30). As of 2019, only one psychiatrist was employed by the Central Penitentiary Administration, who served at the Madrid II Penitentiary Center (29, 46). However, in the absence of official data to reference, healthcare professionals who participated in focus groups estimated that referral psychiatrists spent around three hours per week in those penitentiary centers with more visitation needs. This estimate amounted to less than 7.5% of a full-time work schedule, which typically involves 40 hours per week. **Supplementary Table 7** provides a summary of key dedication estimates based on information shared by focus group participants, categorized according to prison size (small, medium, or large-sized). Interestingly, in 2020, psychiatry emerged as the second most sought-after specialty in Spanish prisons, trailing behind dentistry/stomatology (30) (**Figure 3**).

#### 3.3.3 Acute psychiatric hospitalization in the traditional penitentiary healthcare model

In 2020, acute psychiatric hospitalization within Spanish ordinary penitentiary centers primarily relied on the general nursing modules operating in the center (**Supplementary Table 4**). Of the 66 ordinary penitentiary centers under the General Secretariat of Penitentiary Institutions management, 64 were equipped with a total of 2,916 nursing beds, while 2 centers lacked nursing services altogether. The national average ratio of nursing beds to inmates was 6.7 per 100 inmates, but there was significant variation among centers. The ratios ranged from 1.2 to 19.8 beds per 100 inmates, with smaller centers (those with fewer than 450 inmates) typically reporting higher ratios compared to the larger ones (23, 26) (**Supplementary Table 7**).

During the same year, the nursing modules in these penitentiary centers recorded a total of 21,504 admissions due to various diseases, with 34% attributed to psychiatric pathology (26). For more severe cases and when no nursing resources were available within the penitentiary center, hospitalizations took place within custody areas or RAUs located in reference general hospitals of the public healthcare system. There were 209 RAUs distributed across 39 reference hospitals, with a maximum total capacity of 294 beds. This meant that 86% of penitentiary centers had a reference general hospital with RAUs, with an average of 0.6 beds per 100 inmates (23, 47) (**Supplementary Table 7**).

In 2020, mental health disorders represented 4% of the total 2,460 hospital discharges reported for the prison population (31). They ranked as the ninth most frequent diagnosis at hospital discharge, following other conditions such as digestive, respiratory, or circulatory diseases (31).

#### 3.3.4 Penitentiary psychiatric hospitals in the traditional healthcare model

Penitentiary psychiatric hospitals are designed to treat individuals who have been declared not fully responsible or not responsible at all for their offenses due to mental health disorders. These hospitals combine psychiatric medical care with correctional measures, and the admission and release of persons in Spain are governed by judicial decisions (48–50).

**Figure 4:**
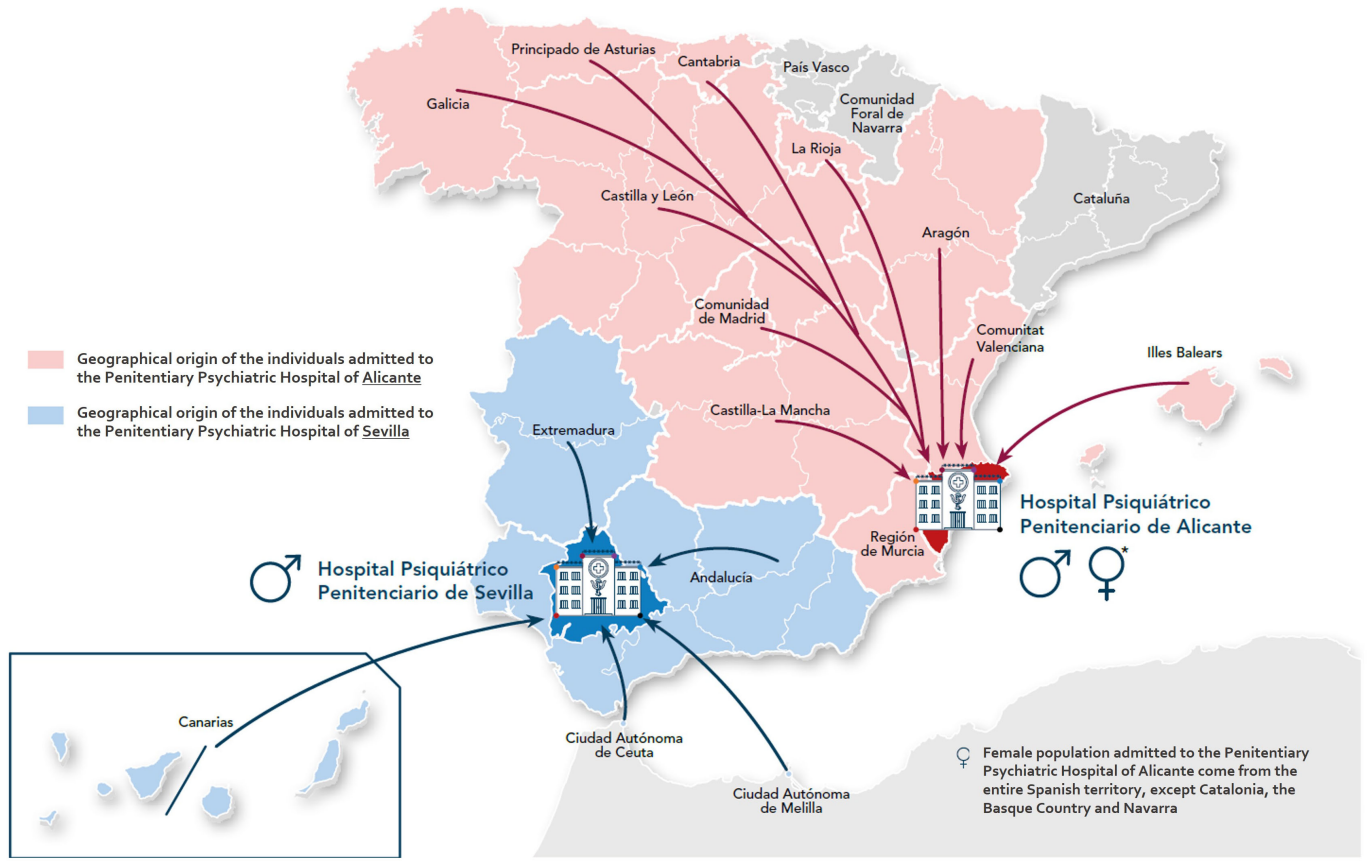
Geographical origin of individuals with SMI admitted to the two penitentiary psychiatric hospitals (in Alicante and Seville) dependent upon the general secretariat of penitentiary institutions (Ministry of Interior) (50, 51). For the preparation of this map, the Spanish official names of the regions were used. Important note: All presented data are estimates of a specific moment that pretend to reflect the status of the situation and trends at the time of data collection. Data should be interpreted with caution, and the status should be verified at each point in time.

The General Secretariat of Penitentiary Institutions operates two penitentiary psychiatric hospitals, located in Alicante (caring for a mixed population of males and females inmates) and Seville (caring for only males) (50, 51). In 2021, these hospitals provided care for a total of 573 inmates, with admissions originating from various parts of Spain, excluding Catalonia and the Basque Country, which manage their own prison healthcare provision (24) (**Figure 4**). The location of these hospitals raises concerns about treatment proximity, a factor recognized as crucial in the recovery and rehabilitation of individuals with SMI (52).

The psychiatric hospitals in Alicante and Seville distinguish themselves from standard hospitals by having psychiatrists fulfill dual roles as clinical caregivers and medico-legal experts, with their organization overseen by penitentiary professionals (53). Consequently, a penitentiary approach to care takes precedence over a healthcare approach (51). A shortage of staff was reported in 2020, with 0.8 psychiatrists per 100 inmates in Alicante and 2.6 in Seville (23).

### 3.4 Innovative mental healthcare models for the incarcerated population with SMI in regions with penitentiary healthcare responsibilities

#### 3.4.1 The Basque country experience

In 2011, the Basque Country government assumed responsibility for healthcare in Basque prisons, committing to uphold inmates’ rights, ensure access to quality healthcare, and promote equitable access, including medical-legal activities (54). One significant challenge was addressing inmates’ mental health needs due to high prevalence, limited resources, and complex organizational structures with unclear medical and legal competencies within prisons (55).

The transfer of prison healthcare from the General Secretariat of Penitentiary Institutions to *Osakidetza*, the Basque public healthcare service provider, involved 43 professionals across three Basque penitentiary centers serving 1,493 inmates (55). Healthcare professionals were integrated into *Osakidetza* with the same status as their counterparts outside prisons. Penitentiary healthcare center managers were appointed, healthcare roles and responsibilities were defined, and procedures for covering vacancies were established (55).

Penitentiary centers were transformed into healthcare facilities equivalent to community health centers (56), adopting the same names with “PC” for “penitentiary center” added to their denominations. Each center was linked to a reference hospital and had access to the full medical resources of the three provinces in the Basque Country (57).

#### 3.4.2 A mental health unit within the penitentiary center

The mental healthcare model adopted at the Zaballa penitentiary center in the Basque Country involves a mental health unit, affiliated with *Osakidetza*, featuring specialized care teams that undertake preventive, diagnostic, and therapeutic interventions, as well as actionable programs directed towards operationalizing the mental health unit within the penitentiary center and with resources in the community outside the prison (**Figure 2B**) (55, 58, 59).

In 2023, the Mental Health Unit at the Zaballa Penitentiary Center consists of a full-time specialized team (**Supplementary Tables 4**, **8**). This team undergoes continuous training within the unit and develops specific skills through on-the-job experience to address the unique challenges of the penitentiary environment. The work of the multidisciplinary team in the Mental Health Unit is organized into six interconnected workstream areas. These areas are designed to address critical actions. They aim to meet the immediate mental health needs of inmates, such as suicide prevention and promoting appropriate prescribing habits of psychotropic drugs. They also focus on promoting seamless coordination among healthcare, legal, and correctional teams within the penitentiary center and in the community. This coordination aims to enhance healthcare management, improve outcomes for inmates, secure the adequate management of health-related information and facilitate continued care in the community after release from prison.

At the heart of the model’s success is the establishment of effective collaboration between the professional teams in the penitentiary Mental Health Unit, the penitentiary primary care center, both under the purview of *Osakidetza*, which handles the healthcare responsibilities, and the penitentiary treatment board, which undertakes disciplinary roles. These three teams collectively address the various aspects of inmates’ lives, including their health and compliance with their sentences in the correctional environment.

This model represents an intermediate level of specialized psychiatric care provision (**Figure 2B**). It is more comprehensive than the traditional penitentiary model, where psychiatrists make specific interventions on an averageable weekly basis (common in most ordinary penitentiary centers in Spain dependent upon the General Secretariat of Penitentiary Institutions). However, it is less complex than models centered around penitentiary psychiatric hospitals seen, for example, in England (51, 60) or the integrated, community care orientated model seen in Barcelona in Catalonia.

Among the medium to long-term psychiatric hospitalization options for judicialized individuals, the 2023-2028 Strategy for Mental Health in the Basque Country (61) describes a range of specific resources. There are acute units located within general hospitals to provide intensive care. Persons with SMI may be admitted urgently through emergency services or scheduled from other care structures, such as the Mental Health Unit in the Zaballa penitentiary center. Stays are typically tailored to individual treatment needs, including diagnostic procedures, detoxification from substances, and attention from various specialists (61). In Gipuzkoa, there is a subsidized penitentiary psychiatry unit at Aita Menni Hospital in Mondragon that functions as a judicial hospitalization facility, admitting individuals with confirmed SMI under court-ordered security measures, offering long-term care for extended psychiatric needs (51, 62) (**Supplementary Table 4**).

Subacute units, situated in psychiatric hospitals, cater to persons needing longer-term treatment, typically lasting from 1 to 3 months. Additionally, rehabilitation units in psychiatric hospitals offer extended care for persons with severe and chronic mental disorders, with the average length of stay estimated at 365 days. Intermediate care devices, such as mental health day hospitals, aim to prevent relapses and promote early discharges, providing intensive care for patients without requiring full-time hospitalization once released from prison. Residential centers and supported housing options cater to individuals with psychiatric disorders requiring ongoing assistance and stimulation for activities of daily living (61).

The continuity of social and health care in the community post-release of individuals with SMI has been crucial. The integration of Health Management Units, streamlining administrative referrals across Basque provinces, has successfully bypassed administrative barriers for primary care in the community. This allows for direct referrals of individuals within mental healthcare resources across the three Basque provinces. Nonetheless, similar to other regions in Spain, resources often fall short in meeting the long-term health and social needs of these individuals in the community.

### 3.5 The Catalan experience

In 2021, the total number of inmates in the Catalan penitentiary system was 7,746, a figure that has remained fairly consistent through 2023 (63). The lifetime prevalence of mental health and SUD among inmates in a Catalan penitentiary setting at the end of their sentence has been estimated at 81.4%, with SUD and attention deficit/hyperactivity disorder (ADHD) being the most common diagnoses (51.4% and 31.4%, respectively) (56). The current prevalence of mental health and SUD is 59.0% (56).

#### 3.5.1 Key feature of the Catalan penitentiary mental healthcare model: comprehensive, individualized, community orientated

Following the transfer of healthcare responsibilities to the Catalan Health System, comprehensive agreements and targeted initiatives improved mental health services, integrating them into the public healthcare network (64–67). By 2017, political efforts emphasized enhancing mental healthcare in prisons to ensure equitable access to community-based care, prioritizing the well-being of incarcerated individuals (68–75).

As a result, Catalonia’s current penitentiary healthcare model is well-established, encompassing in-prison psychiatric services with beds, hospitalization, and acute care units, along with rehabilitation units providing individualized monitoring and continuity of care in the community (**Figure 2C**). The specialized Mental Health Units for hospitalization and rehabilitation, and the Mental Healthcare Programs for continued care in the community are pivotal elements of the model (**Supplementary Table 9**).

The Penitentiary Psychiatric Hospitalization Unit of Catalonia (PPHU-C) operates as a mixed unit, providing diagnosis and treatment to individuals of all genders under judicial supervision, regardless of their legal status. The unit focuses on those who can benefit from specific programs within its purview (70). It functions as a high-security unit where admission is based solely on medical criteria, with discharge determined by both medical and legal considerations (70). Serving as a supra-sectoral resource, the PPHU-C centralizes Catalonia’s penitentiary mental health services and accommodates a total of 66 inpatient beds. It houses various services and units, each with specific objectives tailored to the required level of care (**Supplementary Table 10**). Following psychiatric diagnosis, a comprehensive individualized therapeutic plan (ITP) is devised based on the patient’s specific needs (70).

The Psychiatric Hospitalization and Intensive Rehabilitation Unit of Catalonia (PHIRU-C) stands as a highly specialized unit in penitentiary mental health rehabilitation, catering to the entire Catalan incarcerated population with mental disorders (**Supplementary Table 10**) (69, 75). It adopts a multidisciplinary, recovery-focused approach organized into a continuum of two programs: the Rehabilitation Program which focuses on skill recovery, functional enhancement, and symptom stabilization, and the Community Transition Program, which supports the individual’s reintegration into civilian life. The PHIRU-C works alongside other outpatient services within Catalonia’s sociosanitary care model for judicialized individuals with mental health issues.

Within the community, the Individualized Support Program (ISP) ensures continuity of care through a clinical-social model with intensive follow-up. It assigns a professional case manager to coordinate a tailored therapeutic plan for each individual. In the penitentiary setting, the ISP aims to provide seamless care for individuals with SMI who are at high risk of social exclusion, preventing the onset and persistence of mental health problems (69, 76, 77).

The Primary Care Support Program (PCSP) integrates health and social resources for preventive, proactive, and community-based care. It fosters collaboration between Primary Care Centers (PCC) and Adult Mental Health Centers (AMHC) to enhance mental health care provision. A specialized team, including a psychiatrist, psychologist, and nurse, provides direct care at PCCs, focusing on early detection of decompensation and prevention of self-harm or suicide-related behaviors. The PCSP also offers training and technical support to primary care teams in the community. Each penitentiary center has a dedicated PCSP team collaborating with the regional mental healthcare service provider (73, 78).

The Program for Mental Health Ambulatory Care (PMHAC) is a community-based, multidisciplinary team serving outpatients aged 18 and above with mental disorders. It ensures care continuity, supports families and the community, and participates in rehabilitation and reintegration strategies. The PMHAC prioritizes assisting primary care professionals, caring for individuals with SMI, and coordinating with the entire healthcare system. The PMHAC network collaborates with various levels of health and social care resources within the community (79).

In this way, mental health interventions in Catalonia’s penitentiary psychiatry setting follow a person-centered recovery approach. This empowers individuals to enhance their own capabilities and life skills to improve their quality of life. Recovery-oriented care emphasizes well-being through self-care and early intervention, promoting the long-term recovery efforts, especially for those with SMI. This approach aims to keep SMI individuals in the community, facilitate their reintegration, maintain social connections, and enhance socio-healthcare quality whenever possible (66).

### 3.6 Challenges and opportunities in penitentiary mental healthcare in Spain: insights from healthcare professionals

Healthcare professionals identified significant challenges and opportunities in the mental health care provided within ordinary penitentiary centers and penitentiary psychiatric hospitals that operate outside the National Health System. In ordinary prisons, they emphasized the need for early detection of mental disorders, the critical role of psychiatrists, managing symptom exacerbations, and ensuring continuity of care upon release (**Table 1**).

The penitentiary psychiatric hospitals faced issues such as lack of coordination, conflicts of interest for psychiatrists, heavy workloads, insufficient staffing, training and supervision. Both settings suffer from disparities in working conditions compared to community healthcare, affecting professional motivation and service quality (**Table 2**). However, aligned with findings from the literature (80), healthcare professionals recognized that imprisonment provides an opportunity to engage with socially disadvantaged individuals who often have difficulty accessing community healthcare services and experience relatively poor health outcomes. This presents the potential for achieving significant health improvements

Conversely, in the Basque Country, healthcare professionals noted improvements after the Basque government’s integration of the penitentiary healthcare system into the regional healthcare system. Benefits included better job coverage, satisfaction, training opportunities, and salary parity, contributing to enhanced healthcare provision and professional growth (**Table 3**). The integration also facilitated data sharing through a unified electronic medical record system, improving the continuity and efficiency of care for inmates, especially those with SMI. These insights highlight the potential benefits perceived by healthcare professionals of integrating penitentiary healthcare into broader regional systems to improve outcomes for this vulnerable population.

**Table 3:** Job and training conditions in Basque country penitentiary centers with integrated healthcare: insights from healthcare professionals in focus groups.

Issue	Implications	Illustrative quotes
**Job satisfaction and stability**	Contentment and challenges with sick leave	“*They [General Practitioners at the penitentiary center] are content with their job, and over the years, despite being eligible for transfers based on their seniority and qualifications, they have seldom requested or made such transfers. When there was a shortage of specialists in family medicine, psychiatry, or nursing, it was primarily due to sick leave. This is consistent with the broader challenge faced by the public healthcare system, which struggles to find replacements for sick leave due to a shortage of available doctors*” [00:58:01.410].
**Specific training**	Impact of training on young physicians	*“The young general practitioner physicians in training who come to practice in the mental healthcare unit are enthusiastic. It’s a driving force for generational turnover, helping them overcome any apprehensions, gain valuable experience, and eventually consider working here professionally. This is of great significance”* [00:59:20.670].

## 4 Discussion

This paper outlines the contrasting models for providing mental health care to inmates with SMI in Spain as of 2023. Using a mixed-method approach, it sheds light on the disparate opportunities available to inmates and penitentiary healthcare professionals based on their location. The analysis reveals that Spain, alongside the United Kingdom, Poland, Germany, France, and Italy, is among the European countries with the highest prison population (81). Additionally, Spain’s proportion of women in its prisons was just above 7% in 2021, which is relatively high compared to other European average of 5.7% for the same year (81). Reported frequencies of SMI in the incarcerated population align with estimates from the 2023 WHO/Europe report on prison health, indicating that mental health disorders were present in 32.8% of the European prison population (82). However, underreporting is likely due to incomplete records of noncommunicable diseases and limited data availability (82).

One key factor contributing to this disparity is whether the handover of penitentiary healthcare responsibilities from the General Secretariat of Penitentiary Institutions at the Ministry of the Interior to regional governments and integration into regional healthcare systems has occurred, as mandated in the early 2000s. The traditional penitentiary model persists in regions that have not sought the assigning of healthcare competencies, covering psychiatric and healthcare services in ordinary penitentiary centers and two penitentiary psychiatric hospitals, impacting over 80% of the inmate population in Spain. In contrast, more innovative models have been implemented in regions with transferred competencies and integrated healthcare systems. As of 2023, this applies to Catalonia (since 1983) the Basque Country (since 2011), and Navarra (since 2021), collectively serving approximately 17% of Spain’s inmate population within their respective territories (54, 68, 83). This disparity leads to unequal access to mental healthcare in the penitentiary setting in Spain, significantly disadvantaging the majority of the incarcerated population with SMI. Consequently, precision in psychiatric diagnoses differs, impacting the mental health treatment received by individuals with a psychiatric disorder, the reliability of prevalence data, and the comparability of the available information across penitentiary centers (84, 85).

The findings described in this paper highlights deficiencies in the prevailing traditional penitentiary mental healthcare model in most Spanish penitentiary centers. These deficiencies encompass a shortage of medical professionals in both ordinary prisons and psychiatric hospitals, insufficient staffing, disparities in working conditions and compensation, limited training and specialization, reliance on pGP for psychiatric care and on general nursing modules for psychiatric hospitalization in ordinary penitentiary centers, and limited access to shared medical records due to the lack of integration into the regional healthcare systems. In 2019 and 2020, the WHO recommended a reference ratio of 1.3 full-time dedicated psychiatrists per 1,000 inmates for the European region to address the needs of SMI inmates (42, 86). However, this target remains far from being met in Spanish regions where penitentiary healthcare provision still relies on the General Secretariat of Penitentiary Institutions. Similarly, the allocation of psychiatric staff in both penitentiary psychiatric hospitals is notably lower than in other European countries such as Germany, France, Italy, and the United Kingdom. In these nations, the ratio of specialist psychiatrists to incarcerated individuals in penitentiary psychiatric hospitals has been estimated to be around 5 (51), despite documented challenges in staff coverage and inconsistent utilization of psychiatric penitentiary hospitalization resources (87–91).

This situation may resemble the challenges faced in penitentiary healthcare systems in other parts of the world where prisoners with SMI lack prompt access to specialist mental healthcare professionals or aftercare services on return to prison from in-patient psychiatric services (92–95). Instead, they may receive care from non-specialist healthcare providers, sometimes with the informal support of peer workers (90). In contrast, countries with larger mental health budgets and more psychiatrists per capita, such as Australia, Portugal, and Germany, provide prisoners with access to comprehensive mental health multidisciplinary care (92) similar to the care offered in Alava in the Basque Country and in Catalonia. Experience in England and Wales highlights the need for sufficient resources to ensure screening, triage, assessment, intervention, and reintegration, which constitute essential elements of prison mental health provision to enhance the well-being of this diverse population (80). Other studies confirm the generally positive change in mental health symptoms during detention when adequate mental healthcare is provided (96).

The insufficiency of psychiatric care within correctional facilities due to limited structural and human resources has been well-documented in Spain since 2004 and beyond (97–100). This shortage mirrors the broader situation outside correctional centers, with Spain exhibiting one of the lowest ratios of psychiatric specialists per 100,000 inhabitants in Europe, standing at just 12 psychiatrists per 100,000 inhabitants. This is in contrast to the regional European average of 18 to 20 psychiatrists per 100,000 inhabitants reported for 2020 (101). Notably, there is considerable variability in the availability of psychiatric specialists among different regions, with the Basque Country and Catalonia boasting the highest ratios at 15 and 13 per 100,000 inhabitants, respectively. This difference is likely attributed to the political importance and incentives given to mental healthcare in these regions. Furthermore, Spain significantly lags behind the European average in terms of the number of psychiatric hospital beds available in the community (101). Additionally, the ratio of primary care physicians operating in the community is notably low, with just 0.8 professionals per 1,000 inhabitants as of 2020 (102). Although similar shortcomings have also been described in France, Germany, Italy and the United Kingdom, available resources seem to be more widely available compared with the scenario that predominates in most parts of Spain (87–91, 103).

The traditional penitentiary healthcare model in Spain which assigns the mental health care of inmates with SMI to ordinary penitentiary centers and two penitentiary psychiatric hospitals in regions without assumed prisons healthcare responsibilities, contrasts with the European Commission’s recommendations for mental health and well-being actions (104). These recommendations advocate for transitioning from institutional to community-based mental health care (105). However, providing care for inmates with SMI in the community requires multidisciplinary teams, comprehensive health and social care networks, recovery-focused care, staff training, digital technology use, suitable housing, sustainable policies, and collaborative relationships (106). This underscores the importance of significant investment and collective commitment (107) as demonstrated by the experiences in the Basque and Catalan regions reflected in this study. Stakeholder involvement is crucial in deinstitutionalization planning and barriers such as low political priority, insufficient funding, consensus gaps, and limited cooperation between health and social sectors have been widely reported (92, 108). Consequently, the development of effective and efficient community-based service networks remains partial in many European countries, and primary mental health care for SMI continues limited (109).

The experience in Italy comprises the deinstitutionalization of the forensic psychiatric system that involved replacing psychiatric hospitals with *Residenze per l’Esecuzione delle Misure di Sicurezza* (REMS) and transitioning to community-based treatment for forensic psychiatric patients (110, 111). By 2019, 30 REMS facilities were established across different regions, designed to house up to 20 patients each with a primary focus on therapy and rehabilitation. Notably, these treatments have a limited duration and do not involve police officers. This shift to the Italian REMS model was prompted by the prior forensic psychiatric hospital system, which faced significant issues such as overcrowding, poor hygiene, inadequate treatment programs, non-therapeutic hospitalizations, the presence of penitentiary police personnel, and potentially indefinite admissions (112). Similar challenges were encountered in the two penitentiary psychiatric hospitals run by the General Secretariat of Penitentiary Institutions in Spain. The implementation of the REMS model has effectively addressed these challenges, marking a positive shift in Italy’s approach to forensic psychiatric care (51, 111).

On the other hand, the mental health intervention model implemented in Emilia-Romagna prisons introduces other innovative aspects. Firstly, it adopts a therapeutic approach modeled on multi-professional treatments commonly offered in Italian adult community mental healthcare services. Secondly, it establishes intramural multi-disciplinary Mental Healthcare Service Teams (MHSTs) dedicated to addressing mental health issues. Thirdly, it promotes a culture of collaboration in planning personalized therapeutic-rehabilitation interventions in conjunction with prisoners, their families, and local social/mental healthcare services, ensuring continuity of care during the individual’s transition between prison and the community. This model aims to be accessible to all prisoners in need, structured across different phases of incarceration including assessment, detention, and release. Contrary to traditional approaches, this model emphasizes the role of a multi-disciplinary team rather than solely relying on psychiatrists, thereby redefining the organizational structure of intramural MHSTs (113).

The Mental Health Unit in the Zaballa penitentiary center established in the Basque Country share common features with a similar model established in England (60). Both units are conceived to provide short-term care and ongoing support to prisoners with acute and complex mental health needs until transfer to a psychiatric hospital or back into the prison system. Planning, development and implementation of both models include clear commitment, political will, budgetary responsibility, and pathways for effective collaboration with mental health community teams and services (60).

The establishment of full-time inpatient units designed for inmates, known as *Unités d’Hospitalisation Spécialement Aménagées* (UHSA), in France, such as the one created at the *Paul-Guiraud de Villejuif* hospital, also resembles the experience described in the Mental Health Unit at the Zaballa penitentiary center. The significant changes in forensic psychiatry services have led to improvements in mental health care access and positive outcomes at the UHSA, including a slight decrease in prison suicide rates, similar to the objectives promoted in the Zaballa’s mental health unit. By 2016, eight UHSAs with 380 beds were operational in France (114), but frequent hospitalization of inmates in general psychiatric hospitals (47% of 5,121 psychiatric inmates hospitalizations) persists (114, 115). This trend may stem from the relatively low capacity of UHSAs compared to the increasing incarcerated population. Additionally, substantial distances of up to 300 km between UHSAs and certain prisons might impede emergency UHSA hospitalization, necessitating reliance on local, reachable, general psychiatric facilities. Limited human resources further complicate prisoner transfers from prisons to UHSAs (114). Despite these hardnesses, a satisfaction survey conducted in two UHSAs indicated inmates’ preference for and contentment with hospitalization within the UHSAs over general psychiatric hospitals (116). These changes in penitentiary mental health care have sparked debates (117, 118). Some argue for a dedicated care system within French prisons, while others advocate for keeping psychiatric teams separate from prison environments (117). In line with this debate, the French system prioritizes the separation between the justice and healthcare systems, emphasizing the importance of caregivers’ professional independence and medical confidentiality. This approach aligns with the delineation of roles and functionalities advocated at the Zaballa penitentiary center mental health unit.

In Catalonia, integrating the penitentiary healthcare system into the public healthcare network, establishing specialized units, and expanding mental health services within penitentiary centers and the community have led to a comprehensive and community-focused approach. This represents a significant shift in prison mental healthcare, emphasizing individual needs, recovery support, and community reintegration. Catalonia’s evolving model showcases commitment to care and rehabilitation for inmates with SMI. Given the diversity and lack of evidence in mental health systems for offenders across Europe (89), the Catalan and the Basque models may offer valuable experiences for other regions.

Overall, the experiences of most Spanish regions, where psychiatric care takes place in ordinary penitentiary centers with limited access to other specialized resources, including the penitentiary psychiatric hospitals, differ significantly from those in the Basque Country and Catalonia. Political will, stakeholder understanding, compromise, and leadership are needed to optimize penitentiary mental healthcare provision. After release from prison, accessible pharmacological interventions are paramount in any prevailing model of mental health care. Long-acting injectable antipsychotic medications (depot) with extended dosing intervals that provide adequate symptom control over long periods once symptoms stabilize and treatment simplifies, significantly enhance adherence in individuals with SMI in the community, This effect remains consistent regardless of the impact of sustained outpatient commitment lasting 6 months or longer (119, 120). Long-acting injectable antipsychotic medications offer crucial treatment for SMI individuals, especially those struggling with daily oral medication or limited healthcare access. Biannual dosing, with relapse rates as low as 3.9% over 2 years, ensures efficacy and safety, reducing relapses and functional impairment (121). Eliminating the need for daily doses may enhance patient acceptance, diminish stigma, and boost self-esteem favoring their permanence in the community. Longer medication intervals particularly suit higher-risk patients, like those with recent-onset schizophrenia or in transitional care or challenging living conditions (122).

Limitations in this study stem from a lack of a more detailed information on the therapeutic services within traditional administrative structures, impeding a comprehensive understanding of their approach. While newer systems in the Basque Country and Catalonia show promise, the absence of reported results and outcome measures prevents a thorough assessment of their effectiveness. Additionally, the study does not incorporate perspectives from individuals, as well as their caregivers, with lived experience of SMI within the prison environment, restricting insight into their attitudes towards the newer systems and their potential impact on well-being. The paper also overlooks the empirical question of whether these changes will result in tangible improvements in the lives of incarcerated individuals with SMI, underscoring a gap in understanding and evaluation that warrants further research.

Regarding the methods used, potential subjective and cognitive biases were mitigated through comparison, contextualization and consensus. Caution is necessary when interpreting quantitative data from official reports and public information requests due to poor data availability, which aligns with the lack of high-quality, systematically collected data in forensic psychiatry reported across many European countries (123, 124).

## 5 Conclusions

In conclusion, a dichotomy persists in Spain where both traditional and innovative models of mental healthcare for inmates with SMI coexist in different regions. This divide primarily hinges on whether the responsibility for managing and delivering healthcare in prisons has been decentralized from the national Ministry of Interior to regional healthcare systems as legally stated. Regrettably, this anomaly begets a glaring imbalance in the availability of healthcare resources, processes, and opportunities, disadvantaging both inmates and healthcare professionals working within the prison system and undermining the quality of penitentiary healthcare in most parts of Spain. The innovative and comprehensive models for providing mental healthcare to inmates with SMI, as exemplified in the Basque Country and Catalonia, are feasible and can serve as blueprints for other regions as they assume responsibility for penitentiary healthcare provision.

## Data Availability

The datasets presented in this study can be found in online repositories. The names of the repository/repositories and accession number(s) can be found in the article/**Supplementary Material**.
