# Supplementary material for: Indifferent minds, broken system: a critical examination of mental health care provision for Spain’s incarcerated population with serious mental illnesses
Source: Front Psychiatry. 2024 Aug 21;15:1340155. doi: 10.3389/fpsyt.2024.1340155 (PMC11372278; doi:10.3389/fpsyt.2024.1340155)
Supplement: Supplementary file 1 [file DataSheet1.pdf]

Manuscript: Indifferent Minds, Broken System: a Critical Examination of Mental Health Care Provision for Spain's Incarcerated Population with Serious Mental Illnesses

Supplementary material

Contents

Supplementary Figure 1. Synthesis of the Methodology Used 2

Supplementary Figure 2. Diagram of the Literature Review Method 3

Supplementary Table 1. Search Strategy for the Comprehensive Literature Review 4

Supplementary Table 2. Sources consulted for data extraction on the prison population and general practice physicians operating in prisons (2018-2021) 5

Supplementary Table 3. Calculations and Assumptions for Quantitative Analysis of Prison Population and Healthcare Resources in Spanish Autonomous Communities without Transferred Healthcare Competencies. 6

Supplementary Table 4. Characteristics of the mental healthcare models for the Spanish incarcerated population with SMI in regions with and without assumed penitentiary healthcare responsibilities 8

Supplementary Table 5. Descriptive Prevalence Studies of Mental Disorders on Random Samples of the Inmate Population in Prisons in Spain 12

Supplementary Table 6. Descriptive Prevalence Studies of Mental Disorders on the Inmate Population Assessed in Penitentiary Psychiatric Clinics or Services in Spain 15

Supplementary Table 7. Resources of the traditional mental healthcare model for the incarcerated population with SMI in regions without penitentiary healthcare responsibilities 17

Supplementary Table 8. Resources of the innovative model of the Basque Country for the incarcerated population with SMI (year 2022) 19

Supplementary Table 9. Resources of the innovative model of Catalonia for the incarcerated population with SMI (year 2021) 21

Supplementary Table 10. Services provided at the Penitentiary Psychiatric Hospitalization Unit and the Psychiatric Hospitalization and Intensive Rehabilitation Unit in Catalonia, year 2021 23

References 24

Supplementary Figure 1. Synthesis of the Methodology Used

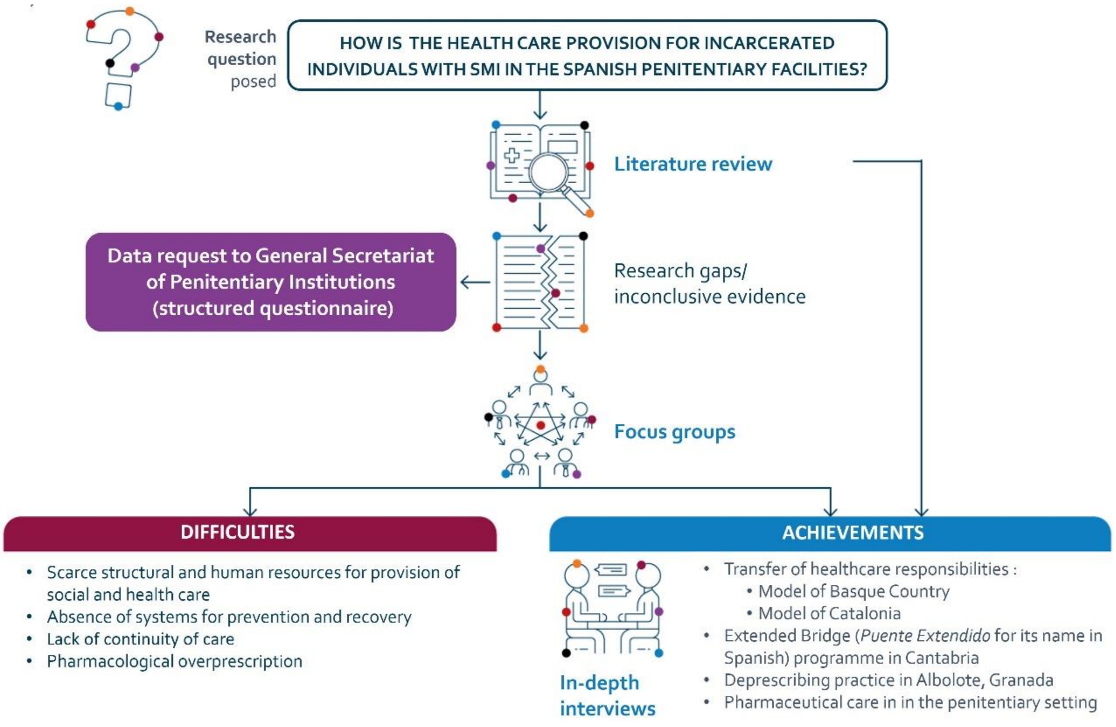

Supplementary Figure 2. Diagram of the Literature Review Method

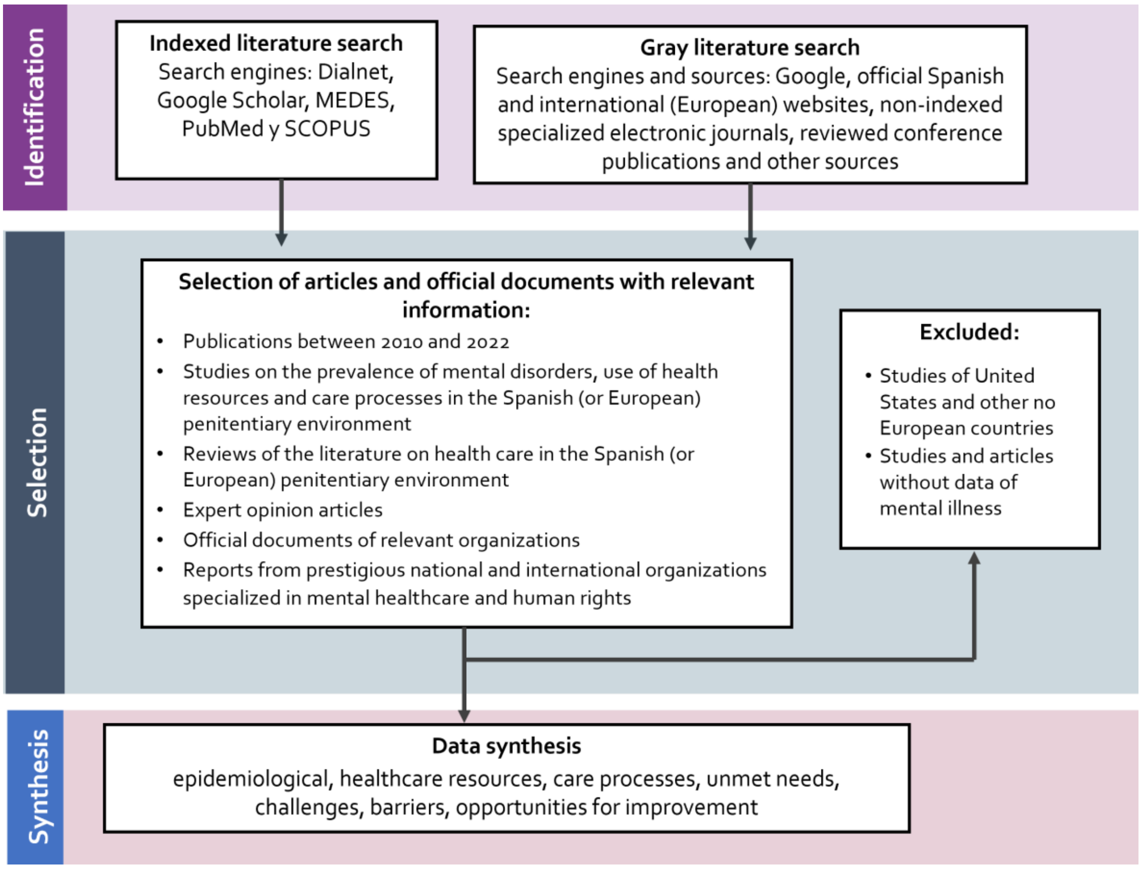

Supplementary Table 1. Search Strategy for the Comprehensive Literature Review

**Scope of the search**	Spain<br>For certain topics, evidence from other European countries has been include
**Search period**	January 2000 - September 2022
**Search engines**	Dialnet, Google Scholar, MEDES, PubMed, and SCOPUS
**Languages**	Spanish, English
**Search terms**	· General Terms: "mental health," "mental disorder," OR "mental illness" OR "psychiatry" OR "prison healthcare" AND "prison" OR "penitentiary center" AND "Spain"<br>· Medical Terms: "schizophrenia" OR "bipolar disorder" OR "major depression" OR "dual diagnosis"<br>· Other specific terms as per the topic.
**Data to be selected and synthesized**	· Epidemiological data<br>· Quantitative data on structural and human healthcare resources<br>· Description of healthcare processes<br>· Needs, barriers, difficulties, challenges, and improvement opportunities
**Topics**	· Definition of SMI<br>· Prevalence of SMI<br>· Healthcare resources for the (mental) health of the prison population<br>· Healthcare process in the care of individuals with serious mental disorders in the prison environment<br>· Programs adapted to individuals with serious mental disorders offered as alternatives to incarceration.<br>· Rational and safe use of psychotropic medications; simplification of pharmacological treatments<br>· Role of the pharmacist in the prison environment<br>· Transfer of penitentiary healthcare competences to the autonomous communities - penitentiary health managed by local/regional healthcare services.<br>· Implementation of an integrated healthcare model<br>· Healthcare for individuals with serious mental disorders in the prison environment in Catalonia
**Main selection criteria**	· Publications, preferably from the most recent decade (2010-2022)<br>· Descriptive and analytical studies with epidemiological data, healthcare resource information, and healthcare process information<br>· Descriptive and analytical studies mainly conducted in Spain and occasionally in other European countries<br>· Literature reviews on healthcare in the prison environment<br>· Expert opinion articles<br>· Official documents from relevant organizations (Ministry of the Interior, Penitentiary Institutions, regional administrations)<br>· Reports from prestigious organizations specializing in mental health and human rights

SMI: Severe Mental Illness

Supplementary Table 2. Sources consulted for data extraction on the prison population and general practice physicians operating in prisons (2018-2021)

Type of data	Sources consulted
**Total prison population**	
**Total prison population by CCAA, as of December 2020 and December 2021**	Statistical data report on the incarcerated population as of December 2020 and December 2021, issued by the General Secretariat of Penitentiary Institutions, Subdirectorate-General of Institutional Relations and Territorial Coordination (1,2).
**Total prison population by prison center, as of December 2020 ***	Report generated by the General Secretariat of Penitentiary Institutions (Ministry of the Interior) in response to the transparency request made by Dr. A. Calcedo on September 5, 2022 (Annex II Primary Care in Penitentiary Institutions: Global and Centers 2020) (3).
**Prison population data for the years 2018 and 2019^**	Prisoner Population Statistics. Criminal, Civil, and Labor Data. General Council of the Judiciary – Spain (4).
**Prison population with high mental health care needs**	
**Prison population with high mental health care needs by CCAA and by prison center, as of July 2019 ***	These data correspond to individuals included in the PAIEM program. Response from the Government on July 9, 2019 (entry 2492) to a written question in Congress 184/284-18/06/2019 by Jon Iñarritu García (GMx), published in the Official Gazette of the Spanish Parliament (5).
**General practice physicians operating in prisons**	
**Prison doctor data for the years 2018 and 2019 ***	Government response (entry 12086) to a written question in Congress 184/1595-23/12/2019 by Jon Iñarritu García (GEHB), published in the Official Gazette of the Spanish Parliament (6).
**Prison doctor data for 2021**	Government response (entry 86386) to a written question in Congress 184/29089-27/11/2020 by Luis Santamaría Ruiz (GP), Isabel María Borrego Cortés (GP), Ana Belén Vázquez Blanco (GP), and Carlos Rojas García (GP), published in the Official Gazette of the Spanish Parliament (7).

CCAA: Autonomous Community; PAIEM (in Spanish, *Programa de Atención Integral a Enfermos Mentales en los centros penitenciarios*): Program of Comprehensive Care for Mentally Ill Patients in Penitentiary Centers; GEHB: (in Spanish: *Grupo Parlamentario Euskal Herria Bildu*): EH Bildu Parliamentary Group; GMx: (in Spanish: *Grupo Mixto):* Mixed Group (in the Spanish Congress of Deputies, the Mixed Group is composed of members who do not belong to a specific parliamentary group due to their parties having a small number of seats: GP: (in Spanish: *Grupo Popular*): People's Party

* These data do not include psychiatric penitentiary hospitals in Alicante and Seville.

^ These data do not include psychiatric penitentiary hospitals in Alicante and Seville. Since data for inmates in psychiatric penitentiary hospitals were not available for the years 2018 and 2019 in the corresponding general reports, an assumption was made using data for inmate populations in the psychiatric penitentiary hospitals in 2020 (151 for the psychiatric penitentiary hospital in Sevilla and 242 for the psychiatric penitentiary hospital in Alicante), which were subtracted from the prison population data of the corresponding years provided by the Judiciary, including the inmate population in psychiatric penitentiary hospitals.

Important Note: Data on medical personnel were extracted from different official sources with unknown data collection and processing methods. Data for 2018 and 2019 report the number of career and interim per prison center, while data for 2021 report the number of deputy medical service directors, medical service chiefs, careers, and interim.

Supplementary Table 3. Calculations and Assumptions for Quantitative Analysis of Prison Population and Healthcare Resources in Spanish Autonomous Communities without Transferred Healthcare Competencies.

Calculated data	Formula	Description of variables
**Prison Population**		
Percentage of **people with high mental health care needs** in relation to the total prison Population# by CCAA	= a/b*100	a: Number of individuals with high mental health care needs by CCAA, corresponding to individuals included in the PAIEM program in July 2019, as per the government's response on July 9, 2019 (entry 2492) to a written question in Congress 184/284-18/06/2019 by Jon Iñarritu García (GMx), published in the Official Gazette of the Spanish Parliament (5).
		b: Total number of individuals by CCAA in July 2019, extracted from the statistical data of the General Council of the Judiciary, Spain. Prison Population Statistics# (4).
**Structural Resources**		
Number of **nursing beds** per 100 individuals by prison center^	= c/d*100	c: Number of nursing beds per prison center as of December 2020, extracted from the general report of Penitentiary Institutions 2020 (8).
		d: Total number of individuals per prison centre for the year 2020, from the report generated by the General Secretariat of Penitentiary Institutions in response to the transparency request (Annex II Primary Care in Penitentiary Institutions: Global and Centers 2020) (3).
Number of **beds in restricted access units** per 100 individuals by CCAA^	= e/f*100	e: Number of beds in restricted access units by CCAA as of December 2020, extracted from the general report of Penitentiary Institutions 2020 (8)
		f: Total number of individuals by CCAA for the year 2020, extracted from the statistical data report on the incarcerated population as of December 2020 issued by the General Secretariat of Penitentiary Institutions, Subdirectorate-General of Institutional Relations and Territorial Coordination (1).
**Human Resources**		
Ratio of **general practice physicians operating in prisons** per 1000 individuals by prison centre or CCAA (2020) ^	= g/h*100	g: Number of general practice physicians per prison center∞ or by CCAA± for the year 2020, from the report generated by the General Secretariat of Penitentiary Institutions in response to the transparency request (Annex II Primary Care in Penitentiary Institutions: Global and Centers 2020) (3).
		h: Total number of individuals per prison centre or by CCAA± for the year 2020, from the report generated by the General Secretariat of Penitentiary Institutions in response to the transparency request (Annex II Primary Care in Penitentiary Institutions: Global and Centers 2020) (3).
Percentage of **medical personnel who left the Penitentiary Institution** compared to total medical personnel	= i/j*100	i: Number of general practice physicians operating in prisons (total, voluntary retirement, or age-related) who left the Penitentiary Institution in 2018, 2019, 2020 or 2021, extracted from the corresponding general reports of the General Secretariat of Penitentiary Institutions (8–11).
		j: Total number of general practice physicians operating in prisons (career, interns, and interim) working in the Penitentiary Institution in 2018, 2019, 2020 or 2021, extracted

Calculated data	Formula	Description of variables
		from the corresponding general reports of the General Secretariat of Penitentiary Institutions (8–11).
Ratio of **specialist psychiatrists** per 1000 individuals by prison center^	= k/h*100	k: Number of specialist psychiatrists per prison centre for the year 2019, extracted from the government's response (entry 12086) to a written question in Congress 184/1595-23/12/2019 by Jon Iñarritu García (GEHB), published in the Official Gazette of the Spanish Parliament (6).
		h: Total number of individuals per prison center◊ for the year 2020, from the report generated by the General Secretariat of Penitentiary Institutions in response to the transparency request (Annex II Primary Care in Penitentiary Institutions: Global and Centers 2020) (3).
Percentage of **full-time dedication of specialist psychiatrists** ∆	= m/40 hours per week*100	m: Number of hours of dedication per week of the inter-consultant doctor/psychiatrist obtained from experiences reported by the healthcare personnel of participating penitentiary centers in the focus groups.
Percentage of **psychiatric resource** usage (*specialized psychiatry consultations*§, *nursing admissions for psychiatric disorders, or hospital discharges following admission for mental disorders*) compared to total specialized consultations, overall and by CCAA^	= n/o*100	n: Number of uses of psychiatric resources (specialized psychiatry consultations, nursing admissions for psychiatric disorders, or hospital discharges following admission for mental disorders) for the year 2020, extracted from the report generated by the General Secretariat of Penitentiary Institutions (Ministry of the Interior) in response to the transparency request (3,12,13)
		o: Total uses of the resource (total specialized consultations, total nursing admissions, or total hospital discharges) extracted from the report generated by the General Secretariat of Penitentiary Institutions (Ministry of the Interior) in response to the transparency request (3,12,13)

CCAA: Autonomous Community; PAIEM: Program of Comprehensive Care for Mentally Ill Patients in Penitentiary Centers

These data do not include psychiatric penitentiary hospitals in Alicante and Seville. Since data for inmates in psychiatric penitentiary hospitals were not available in the annual report of the General Secretariat of Penitentiary Institutions for 2019, an assumption was made using the data for inmate populations in the psychiatric penitentiary hospitals in 2020 (151 for the psychiatric penitentiary hospital in Sevilla and 242 for the psychiatric penitentiary hospital in Alicante), which were subtracted from the prison population data of the Judiciary in July 2019, including the inmate population in psychiatric penitentiary hospitals.

^ These data do not include psychiatric penitentiary hospitals in Alicante and Seville.

+ Only the number of existing beds in restricted access units was counted.

∞ The ratios by prison center were grouped according to the size of the prison population of each center following the distribution: large centers: >1000 inmates; medium centers: 450-1000 inmates; small centers (<450 inmates).

± The data obtained was disaggregated by prison center, so for the calculation by CCAA, the data for each CCAA's prison centers were summed.

◊ Since population data by prison center for the year 2019 was not available, the prison population data for the year 2020 was used, assuming that the prison population is similar.

∆ It is assumed that full-time dedication in one week (100%) corresponds to 8 hours per day for 5 days a week (40 hours per week).

§ For specialized consultations, the percentage of psychiatry consultations by type of consultation (at the prison center, external, or via telemedicine) was also calculated in relation to the total psychiatry consultations using the same source and calculation.

Supplementary Table 4. Characteristics of the mental healthcare models for the Spanish incarcerated population with SMI in regions with and without assumed penitentiary healthcare responsibilities

Characteristics	Without Assumed Responsibilities for Penitentiary Healthcare	With Assumed Responsibilities for Penitentiary Healthcare	
	Traditional model	Innovative models	
		Basque model	Catalan model
Penitentiary healthcare provider	General Secretariat of Penitentiary Institutions	Basque public healthcare services provider (Osakidetza)	Catalan public healthcare services provider (Servei Català de la Salut: CatSalut)
Penitentiary healthcare provision responsibility	Ministry of Interior	Osakidetza<br>(responsibilities for penitentiary healthcare provision assumed in 2011)	CatSalut<br>(responsibilities for penitentiary healthcare provision assumed in 1983)
Management level for penitentiary healthcare provision	National	Regional	Regional
Nature of healthcare management	Penitentiary focused	Public health focused	Public health focused
Regions covered, n	15<br>*Andalucía, Aragón, Asturias, Cantabria, Castilla-La Mancha, Castilla y León, Extremadura, Galicia, Islas Baleares, Islas Canarias, La Rioja, Madrid, Murcia, Navarra*[a]*, Valencia, and Ceuta and Melilla*	1<br>Basque Country	1<br>Catalonia
Penitentiary centers, n	66 PC and across Spain<br><br>Regions with small size, incarcerated population (year 2021)[b]:<br><br>Cantabria (1 PC, 497 inmates) Comunidad Foral de Navarra (1 PC, 346 inmates), Extremadura (2 PC, 970 inmates), and La Rioja (1 PC, 291 inmates)	3 PC [c]<br>Alava province:<br>Zaballa Penitentiary Centre (800 inmates)<br><br>Gipuzkoa province:	10 PC [d]<br>Barcelona province:<br>Brians 1 Penitentiary Center (1,224 inmates)<br>Brians 2 Penitentiary Center (1,898 inmates)<br>Joves Penitentiary Center (388 inmates)

Characteristics	Without Assumed Responsibilities for Penitentiary Healthcare	With Assumed Responsibilities for Penitentiary Healthcare	
	Traditional model	Innovative models	
		Basque model	Catalan model
	Regions with medium size, incarcerated population (year 2021) [b]:<br>Aragón (3PC, 1,783 inmates), Canarias (5 PC, 3,413), Castilla-La Mancha (6 PC, 1,659), Castilla y León (8 PC, 3,419), Galicia (5 PC, 2,818) Iles Baleares (3 PC, 1,388), Principado de Asturias (1 PC, 1,005), and Región de Murcia (2 PC, 1723)<br><br>Regions with large size, incarcerated population (year 2021)[b]:<br>Andalucía (14 PC; 13.220 inmates), Comunidad de Madrid (7 PC, 6.644 inmates) and Comunitat Valenciana (5 PC and penitentiary psychiatric hospital, 6,308)	Martutene Penitentiary Centre (300 inmates)<br><br>Vizcaya province:<br>Basauri-Urbi Penitentiary Centre (260 inmates)	Lledoners Penitentiary Center (1,025 inmates)<br>Quatre Camins Penitentiary Center (1,604 inmates)<br><br>Wad Ras Penitentiary Center (405 inmates)<br><br>Girona province:<br>Puig de les Basses Penitentiary Center (1,014 inmates)<br><br>Lérida province:<br>Ponent Penitentiary Center (582 inmates)<br>Tarragona province:<br>Mas d'Enric Penitentiary Center (1024 inmates)
Total incarcerated population (year 2021), n (%)[b]	45,963 (82.15)	1,388 (2.51)	7,746 (15.40)
Estimated incarcerated population with SMI (year 2021), n (%)	1,834 (4.33)[e]	60 (4.33)<br>56 - 63 (4.00 – 4.50) [f]	336 (4.33)<br>310 – 349 (4.00 – 4.50) [f]
Model of care for SMI	Traditional<br><br>GPs working in the Penitentiary Center providing regular mental healthcare; part-time consultant psychiatrist and/or upon request; acute psychiatric	Innovative + Traditional (depending on the province being considered)<br><br>Araba province: Mental Health Unit in the Zaballa Penitentiary Center linked	Innovative<br><br>Comprehensive mental healthcare and rehabilitation network encompassing hospitalization

Characteristics	Without Assumed Responsibilities for Penitentiary Healthcare	With Assumed Responsibilities for Penitentiary Healthcare	
	Traditional model	Innovative models	
		Basque model	Catalan model
	hospitalization at penitentiary centre nursing general beds or at referral general hospitals with/without RAU<br><br>Psychiatric hospitalization at two penitentiary psychiatric hospitals (providing coverage PC cross country) in Alicante and Sevilla, respectively	to the reference psychiatric hospital for acute hospitalization and to the Araba mental health care network for continued care in the community upon release.<br><br>Gipuzkoa and Vizcaya provinces: GPs working in the PC providing regular mental healthcare; part-time consultant psychiatrist and/or upon request; acute psychiatric hospitalization at referral general hospitals.<br><br>In Gipuzkoa, psychiatric hospitalization can also happen at the Legal Psychiatry Unit Aita Meni	resources: PPHU-C, PHIRU-C; ambulatory care resources: ISP; PCSP; PMHAC

Important note: All presented data are estimates of a specific moment that pretend to reflect the status of the situation and trends at the time of data collection. Data should be interpreted with caution and the current status should be verified.

[a] Navarra is included in the group of regions that have not yet assumed responsibility for their penitentiary healthcare because at the time of reporting these research findings, the traditional model of penitentiary healthcare provision still prevails, as Navarra only acquired these responsibilities in 2021.

[b] Prison population data corresponding to the year 2021, extracted from the National Statistical Data Report on the prisoner population for December 2021 issued by the General Secretariat of Penitentiary Institutions (2)

[c] Data provided by the healthcare professionals from Basque PC who participated in the focus groups celebrated between May and July 2022.

[d] Data on penitentiary population per penitentiary center was not available; data corresponding to the capacity of ordinary penitentiary centers extracted from the statistical data on penitentiary services of the Department of Justice, Rights and Memory of Catalonia (14).

[e] SMI population data correspond to data of the PAIEM (*Programa de Atención Integral al Enfermo Mental*) program and is extracted from the 2021 General Reports of the General Secretariat of Penitentiary Institutions (11)

[f] It is assumed that a similar 4.33% calculated for the country average (or a range of 4% to 4.5% as reported in the literature) applies to the Basque Country and Catalonia.

[g] It is assumed similar figures of prevalence to the one reported for most regions of the country (traditional model)

Characteristics	Without Assumed Responsibilities for Penitentiary Healthcare	With Assumed Responsibilities for Penitentiary Healthcare	
	Traditional model	Innovative models	
		Basque model	Catalan model
GPs: General Practitioners; ISP: Individualised Support Programme; PC: Penitentiary centres; PCSP: Primary Care Support Program; PHIRU-C: Penitentiary Psychiatric Hospitalization and Intensive Rehabilitation Unit of Catalonia; PMHAC: Program for Mental Health Ambulatory Care; PPHU-C: Penitentiary Psychiatric Hospitalization Unit of Catalonia; RAU: Restricted Access Units; SMI: Severe Mental Illness			

Supplementary Table 5. Descriptive Prevalence Studies of Mental Disorders on Random Samples of the Inmate Population in Prisons in Spain

Reference	Design of the study	Location of prisons	Study population, n	Prevalence results on the inmate population
**General Secretariat of Penitentiary Institutions** (15)	Epidemiological study, descriptive, cross-sectional based on clinical history	64 penitentiary centers throughout the Spanish territory	Random sample of 1,009 inmates (92% men, 8% women)	• 3.4% had a psychotic disorder<br>• 12.8% had an affective disorder<br>• 9.4% had a personality disorder<br>• 12.1% had dual pathology<br>• 30.6% of patients were prescribed psychotropic drugs, reaching 47.2% when including methadone<br>• 49.6% had one or more psychiatric diagnoses in their clinical history, including substance abuse or dependence
**Vicens 2011** (16)	PRECA study (first study of this kind in Spain)	Epidemiological study, descriptive, cross-sectional, multicenter	5 prisons in Catalonia, Aragón, and Madrid	Prevalence of mental disorders over the individual's lifetime: 84.4%<br>• 41% had mood disorders (including 22.3% with major depression)<br>• 10.7% had psychotic disorders<br>• 1.8% had bipolar disorder<br><br>Prevalence of mental disorders in the last month: 41.2%<br>• 14.9% had mood disorders (including 7.8% with major depression)<br>• 4.2% had psychotic disorders<br>• 1.0% had bipolar disorder
**López 2016** (17)	Descriptive cross-sectional, multicenter study	2 penitentiary centers in Andalusia (Albolote in Granada and Morón in Seville)	Random sample of 472 inmates	Lifetime prevalence of mental disorders: 82.6%<br>• 31.4% had mood disorders<br>• 11.9% had psychotic disorders<br>Prevalence of mental disorders in the last month: 25.8%

Reference	Design of the study	Location of prisons	Study population, n	Prevalence results on the inmate population
				• 9.3% had mood disorders<br>• 3.8% had psychotic disorders
**Zabala-Baños 2016** (18)	Epidemiological, descriptive, cross-sectional, multicenter study	3 penitentiary centers (2 centers in the Community of Castilla-La Mancha and 1 center in Madrid)	Random sample of 184 inmates	Lifetime prevalence of mental disorders: 90.2%<br>• 72.3% had mental disorders related to substance abuse or dependence<br>• 38.5% had mood disorders (including 28.2% with major depression)<br>• 34.2% had psychotic disorders (11.4% had schizophrenia)<br>• 11.3% had bipolar disorder (I, II, and other)<br><br>Prevalence of mental disorders in the last month: 52.2%<br>• 20.7% had psychotic disorders (9.8% had schizophrenia)<br>• 18.5% had mental disorders related to substance abuse or dependence<br>• 13.0% had mood disorders (including 8.7% with major depression)<br>• 3.2% had bipolar disorder
**Galán Casado 2017** (19)	Descriptive, cross-sectional, multicenter study based on a questionnaire	31 penitentiary centers in 13 Autonomous Communities	Random sample of 310 women in open regime (30.1% of the female penitentiary population)	• 73.2% of women reported having experienced some perceived mental health symptoms<br><br>The most frequently reported symptoms:<br>• 44.8% reported depressive states and 42.9% reported anxiety<br>• 42.3% reported cognitive disorders (lack of concentration and forgetfulness)<br>• 19.4% had dual pathology<br>• 13.2% reported situations related to suicide attempts, and 11% had self-injury processes

Reference	Design of the study	Location of prisons	Study population, n	Prevalence results on the inmate population
				• 12.6% described obsessions/compulsions related to schizophrenia, and 10% reported auditory and persecutory hallucinations
**Arnau 2020** (20)	Multicenter epidemiological, descriptive, and prospective study	3 penitentiary centers in the Valencian Community (Castellón-I, Castellón-II, and Valencia-II)	1,328 inmates	• 81.6% had dual pathology<br>• 68.2% had a cluster B personality disorder<br>• 14% had an affective and/or anxiety disorder<br>• 13% had schizophrenia<br>• Polypharmacy was common (46.6% of patients with regimens of 3-5 medications)

Supplementary Table 6. Descriptive Prevalence Studies of Mental Disorders on the Inmate Population Assessed in Penitentiary Psychiatric Clinics or Services in Spain

Reference	Study Design	Location of penitentiary centres	Study population	Prevalence results on the inmate population attending a psychiatric consultation
**Calvo Estopiñan 2008** (21)	Descriptive, Retrospective, Cross-Sectional Study	Psychiatry Service in the Penitentiary Module of the Miguel Servet University Hospital in Zaragoza	136 inmates admitted to the Penitentiary Module under the Psychiatry Service	The most prevalent primary psychiatric diagnoses were (ICD-9):<br>• 22.0% had personality disorders<br>• 16.3% had schizophrenic disorders
**Arnau-Peiró 2012** (22)	Multicenter Epidemiological Study, Descriptive, Longitudinal, and Prospective	Two prisons in the Valencian Community	786 inmates assessed in the psychiatric clinic	The reasons for referral to the psychiatric clinic from the Primary Care Service in penitentiary centres were:<br>• 17.2% due to the presence of depressive mood<br>• 11.7% due to suspected psychotic symptoms<br>• 7.8% referred for follow-up of stable inmates with underlying psychiatric conditions:<br>o 31.6% patients with personality disorders<br>o 44% patients with psychotic disorders<br>o 13% with depressive disorders<br>• 1.6% referred for follow-up of inmates included in the suicide prevention program<br><br>According to DSM-IV diagnostic criteria:<br>• 20.8% major depressive disorder<br>• 1.5% bipolar disorder<br>• 11.7% schizophrenia spectrum diagnosis<br>• 59.2% personality disorders (over 90% of cluster B, including borderline, antisocial, narcissistic, and mixed)<br>• 17.8% dual diagnosis
**Marín-Basallote 2012** (23)	Cross-Sectional Study Analyzing Psychiatric Care Demand Over One Year	Puerto I, II, and III Penitentiary Centers in	128 inmates who requested psychiatric assistance	The percentage of severe mental disorders was 46.09%

Reference	Study Design	Location of penitentiary centres	Study population	Prevalence results on the inmate population attending a psychiatric consultation
		Puerto de Santa María (Cádiz)		The most prevalent psychiatric diagnoses according to ICD-10 were:<br>• 35.2%, personality disorders (F60-69)<br>• 25.8% schizophrenia, schizotypal disorder, and delusional disorder (F20-29)
ICD: International Classification of Diseases; DSM: Diagnostic and Statistical Manual of Mental Disorders				

Supplementary Table 7. Resources of the traditional mental healthcare model for the incarcerated population with SMI in regions without penitentiary healthcare responsibilities

Penitentiary healthcare provider	General Secretariat of Penitentiary Institutions, Ministry of Interior
Regions covered, n	15<br>*Andalucía, Aragón, Asturias, Cantabria, Castilla-La Mancha, Castilla y León, Extremadura, Galicia, Islas Baleares, Islas Canarias, La Rioja, Madrid, Murcia, Navarra* [a], *Valencia, and Ceuta and Melilla*
**pGPs, ratios** [b]	
**Total, average (year 2020)**	5.2/1,000 inmates
Small size PC (<450 inmates)	4.0 to 32.3 /1,000 inmates
Medium size PC (450-1000 inmates)	3.4 to 11.1 /1,000 inmates
Large size PC (>1000 inmates)	2.0 to 6.3 /1,000 inmates
**Psychiatry consultants at PC**	
**Total, average (year 2022)**[c]	
Small size PC (<450 inmates)	Visits frequency: Once a month (approximately)<br>Duration: < 8 hours<br>Percentage of full-time dedication[d]: < 5%
Medium size PC (450-1000 inmates)	Visits frequency: Once a week or every two weeks<br>Duration: 2-3 hours<br>Percentage of full-time dedication[d]: 2.5%-7.5%
Large size PC (>1000 inmates)	Visits frequency: Once a week<br>Duration: 2-3 hours<br>Percentage of full-time dedication [d]: 5%-7.5%
**Nursing (general) beds, ratios** [b]	
**Total, average (year 2000)**	6.7 beds per 100 inmates
Small size PC (<450 inmates)	1.2 to 18.3 beds per 100 inmates[e]
Medium size PC (450-1000 inmates)	2.7 to 10.1 beds per 100 inmates
Large size PC (>1000 inmates)	4.8 to 9.4 beds per 100 inmates
**Referral general hospitals with RAU** [f]	
**Total, average (year 2020)**	39 general referral hospitals with 294 beds in RAU<br>Ratio[b]: 0.6 beds/100 inmates

Regions with small size incarcerated population (< 1000 inmates) [g]	0.6 to 1.8 beds per 100 inmates
Regions with medium size incarcerated population (1000- 5000 inmates) [h]	0.2 to 1.2 beds per 100 inmates
Regions with large size incarcerated population (>5000 inmates) [i]	0.5 to 0.7 beds per 100 inmates

Important note: All presented data are estimates of a specific moment that pretend to reflect the status of the situation and trends at the time of data collection. Data should be interpreted with caution, and the status should be verified at each point in time.

[a] Navarra is included in the group of regions that have not yet assumed responsibility for their penitentiary healthcare because at the time of reporting these research findings, the traditional model of penitentiary healthcare provision still prevails, as Navarra only acquired these responsibilities in 2021.

b Important mote: ratios are calculated based on data corresponding to a specific moment and may vary throughout the year due to significant fluctuations in the number of incarcerated individuals.

c Data provided by the healthcare professionals who participated in the focus groups celebrated between May and July 2022

[d] Assuming that full-time dedication (100%) corresponds to 8 hours per day for 5 days a week (40 hours per week).

[e] The penitentiary centers of Ibiza and Santa Cruz de la Palma did not have nursing beds in 2020

[f] RAU (Restricted Access Units) are custody areas located in reference hospitals of the public healthcare system. Ciudad Autónoma de Ceuta and Ciudad Autónoma de Melilla did not have RAU in 2020.

[g] Cantabria, Comunidad Foral de Navarra, Extremadura, and La Rioja.

[h] Aragón, Canarias, Castilla-La Mancha, Castilla y León, Galicia, Iles Balerares, Principado de Asturias, and Región de Murcia.

[i] Andalucía, Comunidad de Madrid and Comunitat Valenciana.

PC: penitentiary centers; pGPs: penitentiary General Practitioners; RAU: Restricted Access Units

Supplementary Table 8. Resources of the innovative model of the Basque Country for the incarcerated population with SMI (year 2022)

Penitentiary healthcare provider	Osakidetza: Basque public healthcare services provider		
Regions covered, n	1, *Basque Country*		
Province and penitentiary center	Alava province: Zaballa PC	Gipuzkoa province: Martutene PC	Vizcaya province: Basauri-Urbi PC
Model of care for SMI	Innovative: Mental Health Unit at PC	Traditional	Traditional
Specialized psychiatry resources	Specialised psychiatry full-time dedicated team operating at the mental health unit:<br>3 psychiatrists (1 forensic)<br>1 clinical psychologist<br>3 mental health nurses<br>1 nurse supervisor<br>1 social worker<br><br>PAIEM in the PC providing social, educational and rehabilitation care<br><br>Referral psychiatric hospital for hospitalization	1 part-time psychiatrist (dedication: approx. 7h/week)<br><br>1 part-time clinical psychologist (dedication: approx.14h/week)<br><br>PAIEM in the PC providing social, educational and rehabilitation care<br><br>Penitentiary psychiatric hospitalization at the Legal Psychiatry Unit Aita Meni<br>20 beds<br>Max length of stay: 6 months (with options for extra 6 months extension if needed)<br>Dedicated team: psychiatrists, clinical psychologists, nurses, nurse assistants, social workers<br><br>Referral general hospital for penitentiary psychiatric hospitalization (Donostia hospital)	1 part-time psychiatrist (dedication: approx. 7h/week)<br><br>PAIEM in the PC providing social, educational and rehabilitation care<br><br>Referral general hospital for penitentiary psychiatric hospitalization (Basurto hospital)
General healthcare resources in the PC	Full-time dedicated:<br>6 pGPs<br>2 nurses<br>8 nurse assistants<br>Part-time dedicated:<br>1 pharmacist<br>1 social worker<br>1 educator	Full-time dedicated:<br>3 pGPs<br>4 nurses<br>2 nurse assistants	Full-time dedicated:<br>3 pGPs<br>2 nurses

Penitentiary healthcare provider	Osakidetza: Basque public healthcare services provider		
Regions covered, n	1, *Basque Country*		
Province and penitentiary center	Alava province: Zaballa PC	Gipuzkoa province: Martutene PC	Vizcaya province: Basauri-Urbi PC
Others	Osakidetza portfolio of mental healthcare services in the community within the Alava mental health network[a]	Osakidetza portfolio of mental healthcare services in the community within the Gipuzkoa mental health network (with dedicated programmes to SMI in adults and adolescents)[a]	Osakidetza portfolio of mental healthcare services in the community within the Vizcaya mental health network[a]

Important note: All presented data are estimates of a specific moment that pretend to reflect the status of the situation and trends at the time of data collection. Data should be interpreted with caution, and the status should be verified at each point in time.

[a] Mental healthcare services in the community in Alava, Gipuzkoa and Vizcaya provided by Osakidetza (24–26)

PAIEM: Programa de Atención Integral al Enfermo Mental; PC: penitentiary centers; pGPs: penitentiary General Practitioners; SMI: Severe Mental Illness

Supplementary Table 9. Resources of the innovative model of Catalonia for the incarcerated population with SMI (year 2021)

<table>
<tr><td>Penitentiary healthcare provider</td><td colspan="4">CatSalut: Catalan public healthcare services provider</td></tr>
<tr><td>Regions covered, n</td><td colspan="4">1, Catalonia</td></tr>
<tr><td>Model of care for SMI</td><td colspan="4">Innovative: comprehensive, individualized community orientated</td></tr>
<tr><td colspan="5">Specialized psychiatry resources[a]</td></tr>
<tr><td>Hospital care resources</td><td colspan="4">2 penitentiary psychiatric hospitalization units located in Barcelona, with supratentorial services for the entire Catalonia region
Specialized psychiatry full-time dedicated team operating at:
• PPHU-C
Dedicated team
8 psychiatrists
1 part-time primary care physician
52 nurse assistants
26 general and specialized mental health nurses
2 part-time clinical psychologists
7 part-time social education and social integration technicians
2 part-time social workers
2 part-time clerical personnel
• PHIRU-C
9 psychiatrists
5 primary care physicians
22 general and specialized mental health nurses
26 nurse assistants
22 clinical psychologists
2 social education and social integration technicians
2 clerical personnel</td></tr>
<tr><td>Ambulatory care resources per province</td><td>Barcelona province
• Brians 1 PC:
3 psychiatrists
2 clinical psychologists
1 nurse
0.5 occupational therapist
• Brians 2 PC:
2 psychiatrists
2 clinical psychologists
1 nurse
0.5 occupational therapist
• Joves PC:</td><td>Girona province
• Puig de les Basses PC
1 psychiatrist
1 clinical psychologist
1 nurse
1 occupational therapist

ISP team:
1 case manager</td><td>Lérida province:
• Ponent PC
1 psychiatrist
1 clinical psychologist
1 nurse
1 occupational therapist

ISP team:
1 case manager</td><td>Tarragona province:
• Mas d’Enric PC
1 psychiatrist
1 clinical psychologist
1 nurse
1 occupational therapist

ISP team:
1 case manager</td></tr>
</table>

	0.5 psychiatrists<br>0.5 clinical psychologists<br>0.5 nurse<br>• Lledoners PC:<br>1 psychiatrist<br>1 clinical psychologist<br>1 nurse<br>1 occupational therapist<br>• Quatre Camins PC:<br>2 psychiatrists<br>2 clinical psychologists<br>1 nurse<br>0.5 occupational therapist<br>• Wad Ras PC:<br>0.3 psychiatrists<br>0.3 clinical psychologists<br>0.5 nurse<br><br>ISP team:<br>5.5 case managers			

Important note: All presented data are estimates of a specific moment that pretend to reflect the status of the situation and trends at the time of data collection. Data should be interpreted with caution, and the status should be verified at each point in time.

[a]

ISP: Individualised Support Programme; PC: penitentiary centers; PHIRU-C: Penitentiary Psychiatric Hospitalization and Intensive Rehabilitation Unit of Catalonia; PPHU-C: Penitentiary Psychiatric Hospitalization Unit of Catalonia

Supplementary Table 10. Services provided at the Penitentiary Psychiatric Hospitalization Unit and the Psychiatric Hospitalization and Intensive Rehabilitation Unit in Catalonia, year 2021

**PPHU-C**		**PHIRU-C**	
**Psychiatric emergency service**	A 24/7 service for Catalan prisons, including those requiring admission to a psychiatric penitentiary hospital unit.	**Observation unit**	With 5 beds, catering to short-term admissions to meet the mental health needs of inmates at Brians 2 Penitentiary Center who do not necessitate the specialized care of the PHIRU-C.
**Acute patient admission unit**	Offers maximum psychiatric security, with 10 monitored beds and an average stay of 8.5 days (2021).	**Intensive rehabilitation unit**	With 55 beds, for individuals in need of comprehensive mental health care and rehabilitation, who are serving a sentence or a security measure involving psychiatric internment.
**Subacute patient unit**	Comprising 39 beds and 3 monitored individual rooms, the average patient stay is 73.9 days (2021).	**Community transition unit**	With 20 beds, to assist individuals with a mental disorder who are within 2 years prior to release. In this unit, the rehabilitation process is intensified with the aim of helping the individual reintegrate into the community and the territory.
**Early and preventive care unit for mentally ill individuals with criminal behavior**	Providing 8 beds for individuals needing rehabilitative treatment before a final verdict. The average stay is 198.3 days (2021).		
**Intensive women's rehabilitation unit**	With 6 beds, it caters to female inmate rehabilitation in Catalan prisons (6.6% of the inmate population). The average length of stay is 166.9 days (2021). Admission may occur through emergency services or outpatient care services.		

PHIRU-C: Penitentiary Psychiatric Hospitalization and Intensive Rehabilitation Unit of Catalonia; PPHU-C: Penitentiary Psychiatric Hospitalization Unit of Catalonia

References

1. Secretaría General de Instituciones Penitenciarias Subdirección General de Relaciones Institucionales y Coordinación Territorial, Ministerio del Interior - Gobierno de España. Datos estadísticos de la población reclusa. Total Nacional Diciembre 2020. (2020) https://www.institucionpenitenciaria.es/es/estadística-mensual-2020 [Accessed September 5, 2022]
2. Secretaría General de Instituciones Penitenciarias Subdirección General de Relaciones Institucionales y Coordinación Territorial, Ministerio del Interior - Gobierno de España. Datos estadísticos de la población reclusa. Total Nacional Diciembre 2021. (2021) https://www.institucionpenitenciaria.es/es/estadística-mensual-2021 [Accessed September 5, 2022]
3. Secretaría General de Instituciones Penitenciarias. Ministerio del Interior- Gobierno de España. Respuesta a la solicitud por transparencia realizada por el Dr. A. Calcedo, el 5 de septiembre 2022. (Anexo II- Estadística Sanitaria 2020. Atención primaria en instituciones penitenciarias (global y centros). (2022). 1–76 p.
4. Consejo General del Poder Judicial - España. Estadística de la Población Reclusa. *Datos Penal civiles y laborales* (2022) https://www.poderjudicial.es/cgpj/es/Temas/Estadistica-Judicial/Estadistica-por-temas/Datos-penales--civiles-y-laborales/Cumplimiento-de-penas/Estadistica-de-la-Poblacion-Reclusa/ [Accessed September 8, 2022]
5. Secretaría de Estado de Relaciones con las Cortes y Asuntos Constitucionales, Gobierno de España. Respuesta del Gobierno a la pregunta escrita (entrada 2492) al Congreso 184/284-18/06/2019 por Jon Iñarritu García (GMx). *Boletín Of las Cortes Gen* (2019) https://www.congreso.es/entradap/l13p/e0/e_0002492_n_000.pdf [Accessed September 7, 2022]
6. Secretaría de Estado de Relaciones con las Cortes y Asuntos Constitucionales, Gobierno de España. Respuesta del Gobierno a la pregunta escrita (entrada 12086) al Congreso 184/1595-23/12/2019 de Jon Iñarritu García (GEHB). *Boletín Of las Cortes Gen* (2019) https://www.congreso.es/entradap/l14p/e1/e_0012086_n_000.pdf [Accessed September 7, 2022]
7. Secretaría de Estado de Relaciones con las Cortes y Asuntos Constitucionales, Gobierno de España. Respuesta del Gobierno a la pregunta escrita (entrada 86386) al Congreso 184/29089-27/11/2020 de Santamaría Ruiz, Luis (GP); Borrego Cortés, Isabel María (GP); Vázquez Blanco, Ana Belén (GP); Rojas García, Carlos (GP). *Boletín Of las Cortes Gen* (2021) https://www.congreso.es/entradap/l14p/e8/e_0086386_n_000.pdf [Accessed September 7, 2022]
8. Secretaria General de Instituciones Penitenciarias, Ministerio del Interior - Gobierno de España. Informe General de Instituciones Penitenciarias 2020. (2020) https://www.institucionpenitenciaria.es/es/web/home/fondo-documental/publicaciones [Accessed September 8, 2022]
9. Secretaría General de Instituciones Penitenciarias, Ministerio del Interior - Gobierno de España. Informe General de Instituciones Penitenciarias 2019. (2019) https://www.institucionpenitenciaria.es/es/web/home/fondo-documental/publicaciones [Accessed September 8, 2022]
10. Secretaría General de Instituciones Penitenciarias, Ministerio del Interior - Gobierno de España. Informe General de Instituciones Penitenciarias 2018. (2018) https://www.institucionpenitenciaria.es/es/web/home/fondo-documental/publicaciones [Accessed September 8, 2022]
11. Secretaría General de Instituciones Penitenciarias -, Ministerio del Interior - Gobierno de España. Informe General de Instituciones Penitenciarias 2021. (2021) https://www.institucionpenitenciaria.es/es/web/home/fondo-documental/publicaciones [Accessed February 8, 2023]
12. Secretaría General de Instituciones Penitenciarias, Ministerio del Interior- Gobierno de España. Respuesta a la solicitud por transparencia realizada por el Dr. A. Calcedo, el 5 de septiembre 2022 (Anexo I Estadística sanitaria 2020. Atención especializada global y por centros). (2022). 1–68 p.
13. Secretaría General de Instituciones Penitenciarias, Ministerio del Interior- Gobierno de España. Respuesta a la solicitud por transparencia realizada por el Dr. A. Calcedo, el 5 de septiembre 2022. (Anexo IV Ingresos hospitalarios de II.PP. Nacional, CC.AA. y centros 2020). (2022). 1–189 p.
14. Departament de Justícia D i M. Estadistiques. Serveis penitenciaris. *General Catalunya* (2023)

https://justicia.gencat.cat/ca/departament/Estadistiques/serveis_penitenciaris/ [Accessed March 28, 2024]

15. Dirección General de Instituciones Penitenciarias, Ministerio del Interior- Gobierno de España. Estrategia global de actuación en Salud Mental. Madrid. (2007). https://www.sanidad.gob.es/eu/organizacion/sns/planCalidadSNS/boletinAgencia/boletin10/estudio_saludMental_medio_penitenciario.pdf.pdf
16. Vicens E, Tort V, Dueñas RM, Muro Á, Pérez-Arnau F, Arroyo JM, Acín E, De Vicente A, Guerrero R, Lluch J, et al. The prevalence of mental disorders in Spanish prisons. *Crim Behav Ment Health* (2011) 21:321–332. doi: 10.1002/cbm.815
17. López Álvarez M, Saavedra Macías FJ, López Pardo A, Laviana Cuetos M. Prevalencia de problemas de salud mental en varones que cumplen condena en centros penitenciarios de Andalucía (España). *Rev Española Sanid Penit* (2016) 9:76–85.
18. Zabala-Baños M, Segura A, Maestre-Miquel C, Martínez-Lorca M, Rodríguez-Martín B, Romero M, Rodríguez D. Prevalencia de trastorno mental y factores de riesgo asociados en tres prisiones de España. *Rev Española Sanid Penit* (2016) 18:13–24.
19. Galan Casado D, Ramos-Abalos E, Pinazo Á, Añaños Bedriñada F. Salud mental y consumo de drogas en prisiones españolas. Una perspectiva socioeducativa y de género. *Psychol Soc Educ* (2021) 13:85–98. doi: 10.25115/psye.v10i1.3478
20. Arnau F, García-Guerrero J, Benito A, Vera-Remartínez EJ, Baquero A, Haro G. Sociodemographic, Clinical, and Therapeutic Aspects of Penitentiary Psychiatric Consultation: Toward Integration Into the General Mental Health Services. *J Forensic Sci* (2020) 65:160–165. doi: 10.1111/1556-4029.14137
21. Calvo Estopiñán P, Soler González C, Día Sahún JC, Ventura Faci T. [Prevalence of psychiatric disorders on patients admissed by the Psychiatric Service in the Security Area of H.U.M.S. (University Hospital 'Miguel Servet')]. *Rev Española Sanid Penit* (2008) 10:69–72. doi: 10.4321/S1575-06202008000300002
22. Arnau-Peiró F, García-Guerrero J, Herrero-Matías A, Castellano-Cervera JC, Vera-Remartínez EJ, Jorge-Vidal V, Arques-Egea S, Iranzo-Tatay C. Descripción de la consulta psiquiátrica en centros penitenciarios de la Comunidad Valenciana. *Rev Española Sanid Penit* (2012) 14:20–31.
23. Marín-Basallote N, Navarro-Repiso C. Estudio de la prevalencia de trastorno mental grave (TMG) en los centros penitenciarios de Puerto I, II y III del Puerto de Santa María (Cádiz): nuevas estrategias en la asistencia psiquiátrica en las prisiones. *Rev Española Sanid Penit* (2012) 14:80–85. doi: 10.4321/s1575-06202012000300002
24. Departamento de Salud- Osakidetza. Red de Salud Mental de Bizkaia- Cartera de servicios. *Euskadi (Gobierno vasco)* (2024) https://www.osakidetza.euskadi.eus/osi-salud-mental-bizkaia-servicios/webosk00-sabizcon/es/ [Accessed March 28, 2024]
25. Departamento de Salud- Osakidetza. Red de Salud Mental de Gipuzkoa- Cartera de Servicios. *Euskadi (Gobierno vasco)* (2019) https://www.osakidetza.euskadi.eus/red-salud-mental-gipuzkoa-servicios/webosk00-sagipcon/es/ [Accessed March 28, 2024]
26. Departamento de Salud- Osakidetza. Red de Salud Mental de Araba- Cartera de Servicios. *Euskadi (Gobierno vasco)* (2019) https://www.osakidetza.euskadi.eus/red-salud-mental-araba-servicio/webosk00-saaracon/es/ [Accessed March 28, 2024]
